# Deducing the source and composition of rare earth mineralising fluids in carbonatites: insights from isotopic (C, O, ^87^Sr/^86^Sr) data from Kangankunde, Malawi

**DOI:** 10.1007/s00410-017-1412-7

**Published:** 2017-11-09

**Authors:** Sam Broom-Fendley, Frances Wall, Baruch Spiro, Clemens V. Ullmann

**Affiliations:** 1 Camborne School of Mines, University of Exeter, Penryn Campus, Cornwall, TR10 9FE UK; 2 Department of Earth Sciences, Natural History Museum, Cromwell Road, London, SW7 5BD UK; 3 Environment and Sustainability Institute, University of Exeter, Penryn Campus, Cornwall, TR10 9FE UK

**Keywords:** Carbonatites, Rare earth elements, C and O isotopes, Sr isotopes, Chilwa Alkaline Province, Critical metals

## Abstract

Carbonatites host some of the largest and highest grade rare earth element (REE) deposits but the composition and source of their REE-mineralising fluids remains enigmatic. Using C, O and ^87^Sr/^86^Sr isotope data together with major and trace element compositions for the REE-rich Kangankunde carbonatite (Malawi), we show that the commonly observed, dark brown, Fe-rich carbonatite that hosts REE minerals in many carbonatites is decoupled from the REE mineral assemblage. REE-rich ferroan dolomite carbonatites, containing 8–15 wt% REE_2_O_3_, comprise assemblages of monazite-(Ce), strontianite and baryte forming hexagonal pseudomorphs after probable burbankite. The ^87^Sr/^86^Sr values (0.70302–0.70307) affirm a carbonatitic origin for these pseudomorph-forming fluids. Carbon and oxygen isotope ratios of strontianite, representing the REE mineral assemblage, indicate equilibrium between these assemblages and a carbonatite-derived, deuteric fluid between 250 and 400 °C (δ^18^O + 3 to + 5‰_VSMOW_ and δ^13^C − 3.5 to − 3.2‰_VPDB_). In contrast, dolomite in the same samples has similar δ^13^C values but much higher δ^18^O, corresponding to increasing degrees of exchange with low-temperature fluids (< 125 °C), causing exsolution of Fe oxides resulting in the dark colour of these rocks. REE-rich quartz rocks, which occur outside of the intrusion, have similar δ^18^O and ^87^Sr/^86^Sr to those of the main complex, indicating both are carbonatite-derived and, locally, REE mineralisation can extend up to 1.5 km away from the intrusion. Early, REE-poor apatite-bearing dolomite carbonatite (beforsite: δ^18^O + 7.7 to + 10.3‰ and δ^13^C −5.2 to −6.0‰; ^87^Sr/^86^Sr 0.70296–0.70298) is not directly linked with the REE mineralisation.

**Electronic supplementary material:**

The online version of this article (10.1007/s00410-017-1412-7) contains supplementary material, which is available to authorized users.

## Introduction

Carbonatites are characteristically enriched in the rare earth elements (REE) and are host to many of the largest REE deposits (Wall 2014; Verplanck et al. 2016). The processes behind the formation of these deposits are increasingly of interest due to the recognition of the REE as a ‘critical metal’ in terms of its supply security (e.g., Chakhmouradian and Wall 2012; European Commission 2014; Chakhmouradian et al. 2015). Only certain carbonatites, however, are REE-rich [e.g., Mountain Pass and Bear Lodge, USA (Castor 2008; Moore et al. 2015; Verplanck et al. 2016); Kangankunde, Tundulu and Songwe, Malawi (Ngwenya 1994; Wall and Mariano 1996; Broom-Fendley et al. 2016a, 2017a, b); Wigu Hill, Tanzania (Mariano 1989); Khibiny, Russia (Zaitsev et al. 1998, 2002, 2015); Barra do Itapirapuã, Brazil (Andrade et al. 1999; Ruberti et al. 2008); and the Mianning-Dechang and Qinling belts in China (Kynicky et al. 2012)]. It is almost always during the last stages of carbonatite emplacement that REE concentrations become high enough to form significant, sometimes rock-forming, quantities of rare earth minerals (e.g., Wall and Zaitsev 2004; Chakhmouradian and Zaitsev 2012). Initial REE mineralisation takes place near the magmatic–hydrothermal interface. Subsequent redistribution and re-working can be associated with silicification, fluorite crystallisation, vug-infilling and other late-stage processes (Wall and Mariano 1996; Doroshkevich et al. 2009).

The earliest REE mineral thought to crystallise in most REE-rich carbonatites is the Na–Ca–REE–Ba–Sr carbonate, burbankite (Wall and Zaitsev 2004). This mineral forms characteristic hexagonal crystals, either co-genetic with calcite in calcite carbonatites (e.g., Bear Lodge and Shaxiongdong; Xu et al. 2008; Moore et al. 2015) or in later REE-rich pegmatites (e.g., Khibiny; Zaitsev et al. 1998). The primary nature of burbankite is supported by its stable (O and C) isotope composition, which is within the range of values for primary magmatic carbonatites (Zaitsev et al. 2002). However, burbankite is not commonly preserved and, in REE-rich carbonatites, the REE are typically hosted in REE–(fluor)carbonates or monazite, commonly in an assemblage with strontianite and baryte. Partial replacement of burbankite by this mineral assemblage has been recognised at a number of localities where the hexagonal pseudo-structure of burbankite is retained (Zaitsev et al. 1998; Moore et al. 2015). REE-bearing hexagonal pseudomorphs at other REE-rich carbonatites are thus thought to have formed through the same process of burbankite replacement (e.g. Kangankunde, Malawi; Wigu Hill, Tanzania; Gem Park, Colorado; Adiounedj, Mali; Wall and Mariano 1996). Subsequent REE mineralisation is likely to be the result of hydrothermal fluids transporting and redistributing the REE. A significant content of carbonate in these fluids is inferred and they are therefore termed (carbo)-hydrothermal (following Zaitsev et al. 1998). Mineralisation in these examples commonly forms in vugs and/or along veins (e.g., Doroshkevich et al. 2009).

Despite the importance of the transition from burbankite to other REE minerals, little is known about the temperature of this mineral replacement, the source of the fluid or how it evolves during REE redistribution. Stable isotopes (C and O) provide a useful tool to assess the above questions. Many of the effects of the principal petrological processes in carbonatites are reasonably well understood (e.g., Deines 1989; Demény et al. 2004). Based on a stable (C and O) isotope study of burbankite replacement at Khibiny (syn. Khibina) and Vuoriyarvi, Zaitsev et al. (2002) suggested burbankite replacement occurred through open-system, post-magmatic isotopic exchange with a low-temperature, water-rich, fluid. REE redistribution occurs in rocks where associated carbonates exhibit a trend toward ^18^O- and ^13^C-enrichment (e.g., Andrade et al. 1999; Downes et al. 2014; Trofanenko et al. 2016). In a single bastnäsite-(Ce) analysis from Belaya Zima, this enrichment has also been shown in REE mineralisation (Doroshkevich et al. 2016). Depending on the degree of enrichment of ^18^O and ^13^C, this increase has been attributed to Rayleigh fractionation [where δ^18^O and δ^13^C have a correlation coefficient of approximately 0.4 (Deines 1970, 1989; Ray and Ramesh 2000)] or through alteration from successive generations of hydrothermal fluid (Santos and Clayton 1995; Ray and Ramesh 1999). In some carbonatites, more abnormal trends have been noted: for example, at the Songwe Hill, Arshan, Fen and Igaliko carbonatites, decreasing δ^18^O values have been ascribed to either mixing with hot meteoric water, rapid CO_2_ degassing or crystallisation from a low-δ^18^O magma (Andersen 1984; Pearce and Leng 1996; Pearce et al. 1997; Doroshkevich et al. 2008; Broom-Fendley et al. 2016b; Doroshkevich et al. 2016).

In this contribution, we present major and trace element data and the results of a multi-isotope study (C, O, ^87^Sr/^86^Sr) on the REE-rich Kangankunde carbonatite, Malawi. We aim to understand the relationship between the host carbonatite and the REE mineralisation with respect to the role of deuteric (magma-derived) and meteoric water as well as country rock components. For this, we have analysed strontianite, which occurs as part of the REE pseudomorph assemblage, as a proxy for the process of REE mineralisation as well as more conventional analyses of dolomite and calcite. O isotopes are also presented from quartz mineralisation to elucidate the importance of this late phase of more enigmatic REE mineralisation.

## Geological background

**Fig. 1:**
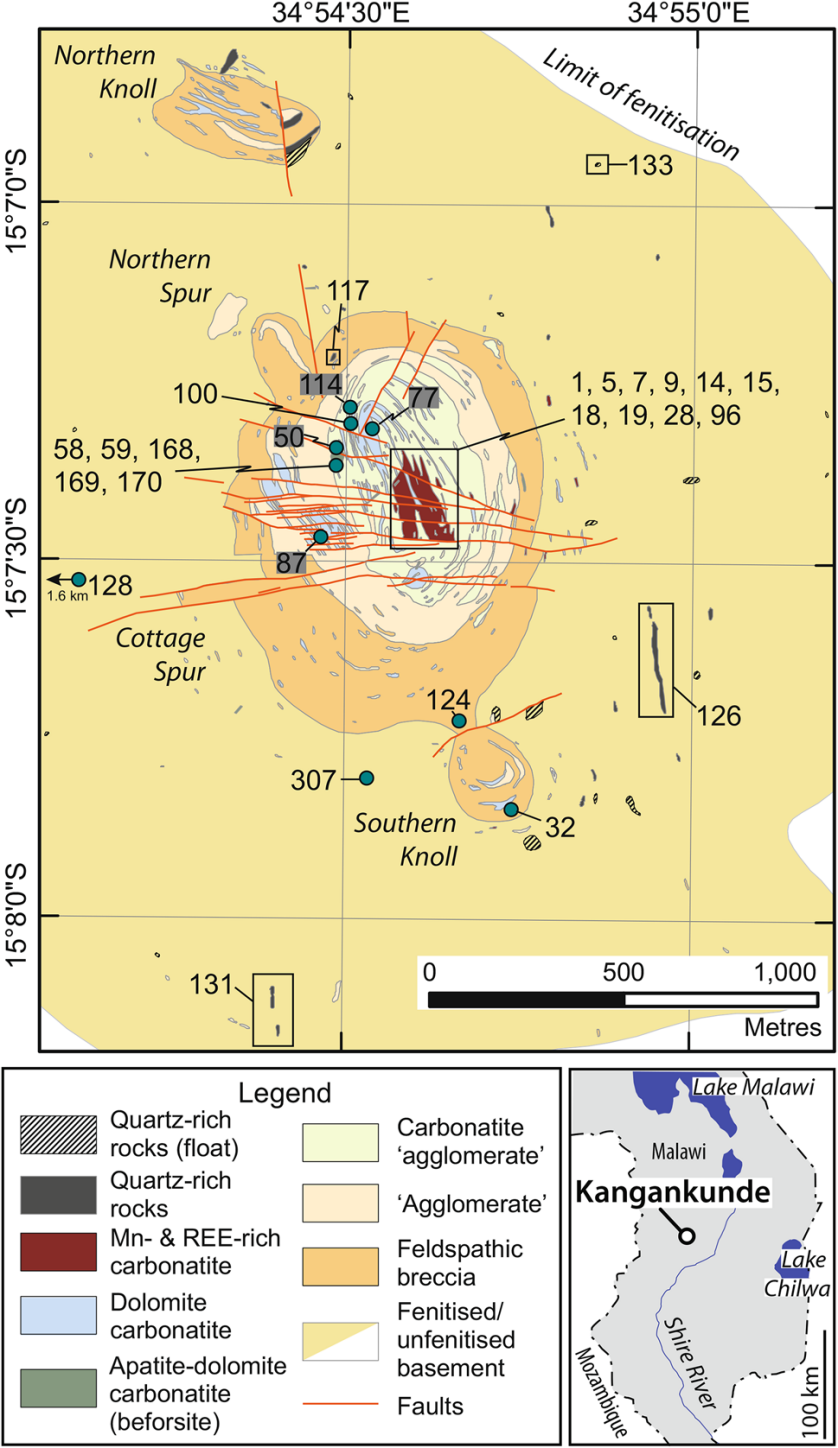
Geological map of Kangankunde showing the approximate sample locations. Samples 1–32 prefixed with BM1993 (P4); 33–133 prefixed with BM1962 (73); 134–306 with SoS-; and 307 with BM1968 (P37). The term ‘agglomerate’ is location-specific and denotes a breccia comprising fragments of country rock and carbonatite in a matrix of carbonatite (see Garson and Campbell Smith 1958). Inset map indicates the position of Kangankunde in southern Malawi. Maps redrawn after Garson and Campbell Smith (1965) and Broom-Fendley et al. (2016a)

The Kangankunde carbonatite is one of the largest in the Chilwa Alkaline Province (CAP) of Southern Malawi: a 200–300 km area comprising Late Jurassic–Early Cretaceous alkaline rocks and carbonatites (Garson 1965; Woolley 1991, 2001). The carbonatite forms a low hill, some 200 m above the surrounding plain, with lower slopes composed of fenitised and, locally, brecciated rocks while the upper slopes are predominantly carbonatite. The earliest intrusion stage at Kangankunde is a small body of apatite–dolomite carbonatite, termed beforsite by Garson and Campbell Smith (1965). This terminology is retained in this manuscript in order to clearly differentiate beforsite from the majority the intrusion, which is formed of arcuate lobes of late-stage, REE-rich, ferroan-dolomite carbonatites, some of which are also Mn-rich (Garson and Campbell Smith 1965; Fig. 1). Clear evidence for the order of intrusion is indicated by local metasomatism of beforsite by REE-bearing fluids and the formation of incipient REE mineralisation in these samples. This consists of increased REE contents in later zones in apatite, and the formation of monazite at corroded apatite crystal margins (Wall and Mariano 1996). The last stage to occur is composed of quartz-rich rocks which predominantly, although not exclusively, occur within the fenite aureole outside of the main intrusion (Fig. 1). This aureole extends up to approximately 2 km from the intrusion and is formed of proximal potassic and distal sodic fenitized Precambrian gneiss (Woolley 1969).

**Fig. 2:**
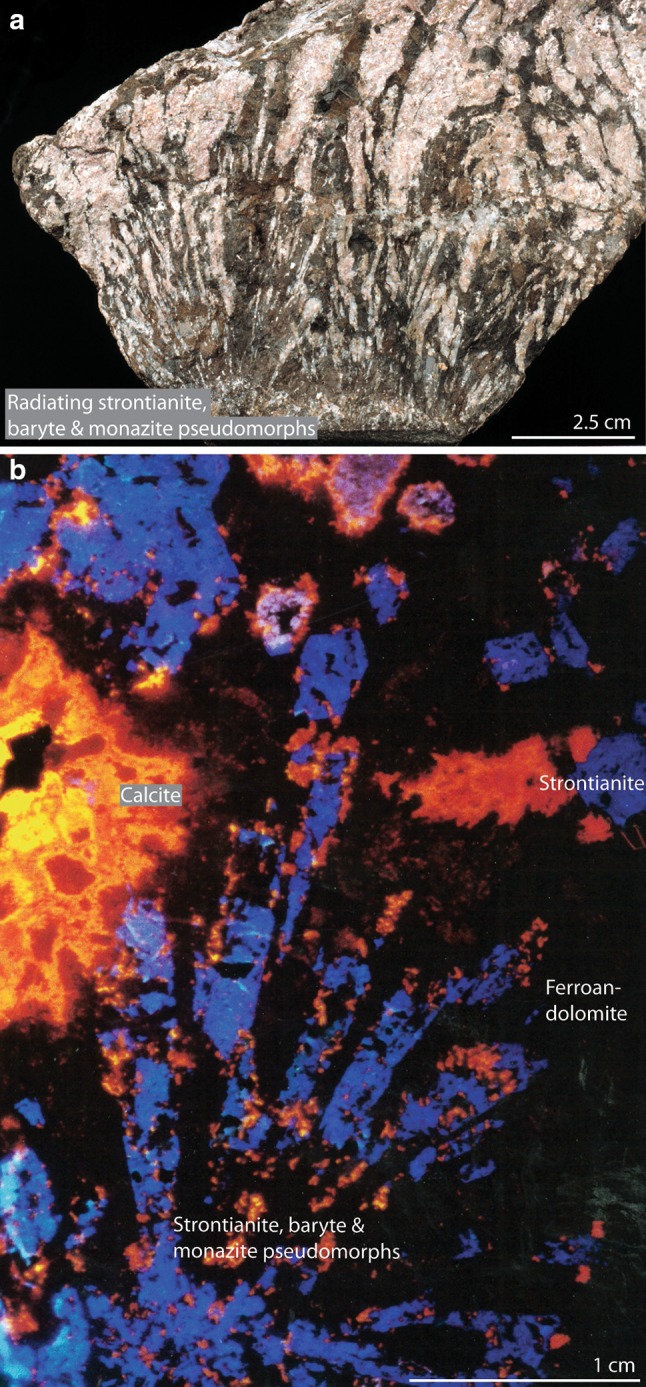
Radiating monazite–baryte–strontianite pseudomorphs in dolomite carbonatite. **a** Sample BM 1962, 73 (107), after Wall and Mariano (1996). **b** Cold-cathodoluminescence image of sample: BM, 1962, 73 (100), courtesy of Tony Mariano

Much of the carbonatite at Kangankunde contains 5–10 wt% REE, which has stimulated historic mineral exploration (e.g. Holt 1965; Dallas et al. 1987) and the usage of Kangankunde samples for a REE remote-sensing feasibility study (Neave et al. 2016). The most abundant REE mineral is distinctive sector-zoned green monazite-(Ce), which has been well studied with respect to its crystal structure and REE uptake (e.g. Ni et al. 1995; Cressey et al. 1999). Bastnäsite-(Ce) and goyazite–florencite-(Ce) are also important REE minerals at Kangankunde (Wall and Mariano 1996). Rare earth minerals invariably occur in an assemblage with baryte, strontianite and, locally, ferroan dolomite and quartz. These minerals occur as spectacular hexagonal pseudomorphs (Fig. 2), but also in veinlets and drusy cavities (Wall and Mariano 1996).

A range of rare earth minerals also occur within the late-stage quartz-rich rocks, including florencite–goyazite and apatite. The latter mineral displays evidence for multiple stages of dissolution–reprecipitation related to late alteration (Wall and Mariano 1996; Broom-Fendley et al. 2016a). The most common REE mineral, however, is monazite occurring in a similar assemblage to that found in the main carbonatite, but without associated strontianite. Wall and Mariano (1996) associate the REE mineralisation in the quartz rocks with the mineralisation in the main carbonatite on the basis of their identical major element composition and REE distribution.

Few isotopic analyses have been carried out on Kangankunde material. ^87^Sr/^86^Sr ratios in strontianite and monazite were measured by Hamilton and Deans (1963) and whole-rock ^87^Sr/^86^Sr and ^144^Nd/^143^Nd ratios were determined in one specimen by Nelson et al. (1988) and Verplanck et al. (2016), and in three specimens by Ziegler (1992). Snelling (1965) dated the intrusion as 123 ± 6 Ma using K–Ar in phlogopite. Wall et al. (1994) calculated a whole-rock Sm–Nd isochron age of 136 ± 11 Ma, and provided an early synopsis of the C and O isotope data presented herein.

## Approach and analytical procedures

The specimens used in this study are those of Garson and Campbell Smith (1965) from the collection hosted at the Natural History Museum, London [BM,1962.73 (50–133)], supplemented by samples collected by the authors [BM,1993.P4 (1–32); SoS168–170] and Alan Woolley [BM,1968.P37 (307)]. Descriptions of the samples are outlined in the supplementary information, summarised in Table 1, and their locations are plotted on Fig. 1. Many of the specimens have been previously described by Garson and Campbell Smith (1965), Woolley (1969) and Wall and Mariano (1996). These samples were re-investigated for this study in order to locate fluid inclusions for microthermometry. Unfortunately, inclusions which are clearly petrographically linked to REE mineralisation do not occur at Kangankunde. Some brief notes on the nature of the inclusions are presented in the supplementary information. Rocks are considered REE-rich when REE_2_O_3_ concentrations are above 2% and REE-poor when below this value (similar to Jones et al. 2013). Samples were selected to represent the range of different varieties of REE-rich carbonatites and contrasting barren rocks at Kangankunde. They are divided into:

**Table 1:** Rock descriptions and carbon, oxygen and strontium results from Kangankunde samples

BM number	Rock			Mineral		Colour		δ^13^C ‰ (VPDB)		δ^18^O ‰ (VSMOW)	^87^Sr/^86^Sr_i_
*Apatite–dolomite carbonatites* (*beforsites*)											
1962,73(50)	Apatite-bearing dolomite carbonatite			Dolomite				− 6.02		7.70	0.702977 (± 6)
				Dolomite				− 6.03		7.79	
				Insoluble residue							0.702973 (± 6)
1962,73(58)	Apatite-bearing dolomite carbonatite			Dolomite				− 5.92		8.20	0.702974 (± 6)
				Insoluble residue							0.702956 (± 6)
1962,73(59)	Apatite-bearing dolomite carbonatite			Dolomite				− 3.28		8.66	
SoS_168	Apatite-bearing dolomite carbonatite			Bulk powder				− 5.56		10.26	
SoS_169	Apatite-bearing dolomite carbonatite			Bulk powder				− 5.82		10.25	
SoS_170	Apatite-bearing dolomite carbonatite			Bulk powder				− 5.20		9.54	
*REE-rich carbonatites*											
1993,P4(1)	REE-rich carbonatite, portion rich in monazite-(Ce)			Ankerite		mlb		− 1.96		7.69	
				Strontianite				− 3.45		4.40	
				Strontianite				− 3.42		4.56	
	REE-rich carbonatite, portion poor in monazite-(Ce)			Ankerite		mlb		− 2.07		8.77	0.703033 (± 5)
				Ankerite		mlb		− 2.07		8.67	
				Dolomite		lb		− 2.51		13.58	
				Strontianite				− 3.42		3.68	0.703048 (± 6)
1993,P4(7)	REE-rich carbonatite with pseudomorphs			Monazite-(Ce)							0.702989 (± 7)
				Ankerite		blk		− 0.98		23.43	
				Ankerite		mdb		− 1.16		21.44	
				Ankerite		mdb		− 0.99		23.71	0.703027 (± 7)
				Ankerite		mdb		− 0.98		23.67	
				Strontianite				− 3.35		4.37	0.703028 (± 6)
				Calcite				− 2.03		17.97	
				Calcite				− 1.74		20.65	
	REE-rich carbonatite without pseudomorphs			Ankerite		blk		− 0.94		23.39	
				Ankerite		mdb		− 1.16		21.48	
				Ankerite		mdb		− 1.02		23.55	
				Ankerite		mdb		− 1.04		23.45	
				Dolomite				− 0.96		23.50	
1993,P4(9)	REE-rich carbonatite			Ankerite		mlb		− 3.68		7.88	0.703030 (± 5)
				Ankerite		mb		− 3.58		11.90	0.703035 (± 5)
				Strontianite				− 3.48		5.00	0.703058 (± 6)
				Calcite				− 7.73		21.39	0.703074 (± 7)
1993,P4(14)	REE-rich carbonatite			Calcite				− 4.09		22.47	
				Calcite				− 4.13		19.13	
				Strontianite				− 3.38		6.72	
				Calcite				− 4.54		13.53	
1993,P4(15)	REE-rich carbonatite			N/A							
1993,P4(19)	Host carbonatite			Ankerite		mlb		− 2.32		11.35	
				Ankerite		mlb		− 2.79		14.62	
				Dolomite		lb		− 3.57		17.75	
				Apatite							0.703034 (± 7)
	Monazite-(Ce)-ferroan-dolomite vein			Ferroan Dolomite		w		− 1.81		7.76	
				Ankerite		w		− 1.85		8.09	
				Monazite-(Ce)							0.703056 (± 7)
1962,73(59)	Vein in apatite-bearing dolomite carbonatite			Dolomite				− 2.52		6.50	
1962,73(77)	Small dark vein			Ankerite		blk		− 2.88		19.63	
	Dark coloured host			Ankerite				− 2.08		16.82	
				Ankerite				− 2.07		16.92	
	REE-rich, light-coloured carbonate			Ankerite		lb		− 2.63		14.10	
				Strontianite				− 3.31		3.32	
1962,73(87)	REE-rich carbonatite			Ankerite		mlb		− 2.37		14.65	0.703067 (± 6)
				Ankerite		lb		− 2.55		16.45	
				Ferroan dolomite		lb		− 3.03		18.50	
				Dolomite		lb		− 4.62		21.35	0.703029 (± 6)
1962,73(100)	REE-rich carbonatite with REE pseudomorphs			Ankerite		w		− 2.64		6.82	
				Ankerite		w		− 2.61		6.72	
				Ankerite		lb		− 2.50		9.07	
				Strontianite				− 3.22		4.86	
				Strontianite				− 3.26		4.79	
				Calcite				− 4.20		9.29	
				Monazite-(Ce)							0.703029 (± 7)
				Quartz						13.2	
*REE-poor carbonatites and veins*											
1993,P4(5)	Carbonatite with little monazite-(Ce)			Ankerite		w		− 2.16		6.62	
				Strontianite				− 3.52		4.10	
1993,P4(18)	Host carbonatite			Ferroan Dolomite		mdb		− 3.51		18.9	
	Vein along joint plane			Calcite				− 6.60		22.68	0.703016 (± 6)
1993,P4(28)	Dark, medium-grained carbonatite			N/A							
1993,P4(29)	Manganiferous, REE-poor carbonatite			Ankerite		mdb		− 1.43		12.52	
				Whole-rock							0.703061 (± 7)
1962,73(96)	Quartz–apatite rock in manganiferous carbonatite			Quartz						9.7	
*Monazite-rich quartz rocks from within the main intrusion*											
1962,73(114)	Monazite-(Ce), florencite, baryte, quartz rock			Quartz						12.9	
				Quartz						12.6	
				Quartz						12.5	
				Quartz						12.9	
				Monazite-(Ce)							0.703029 (± 7)
1962,73(117)	Monazite-(Ce), florencite, baryte quartz rock			Quartz						15.3	
				Quartz						15.7	
				Monazite-(Ce)							0.703020 (± 7)
*Quartz rocks from outside of the main intrusion*											
1962,73(124)		Monazite–quartz rock			Quartz					13.9	
					Quartz					14.5	
					Quartz					14.5	
					Monazite-(Ce)						0.703048 (± 7)
1962,73(126)		Monazite–quartz rock			Quartz					17.0	
1962,73(128)		Quartz, florencite, Fe-oxide rock			Quartz					14.8	
					Quartz					14.9	
					Quartz					14.9	
					Quartz					15.2	
					Whole-rock						0.703423 (± 6)
1962,73(131)		Quartz–apatite–Fe-oxide rock			Quartz					16.9	
					Quartz					17.1	
					Quartz					17.7	
					Apatite concentrate						0.703045 (± 6)
1962,73(133)		Quartz–fluorite rock			Quartz					8.8	
					Whole-rock						0.703423 (± 7)
					Fluorite concentrate						0.703428 (± 7)
*Fenites and country rock gneiss*											
1993,P4(32)			Massive vein quartz	Quartz						9.0	
1968,P37(307) a			Quartz fenite	Quartz						8.3	
				Whole-rock							0.704644 (± 7)^a^
1968,P37(307) b			Fenite vein	Whole-rock							0.703280 (± 7)^a^

Colour data: blk, black; lb, light brown; mlb, mottled light brown; mdb, mottled dark brown; w, white. Full rock descriptions in the supplementary information

^a^1968,P37(307)a: initial value, calculated from Rb 8.97 ppm, Sr 133.30 ppm; ^87^Sr/^86^Sr 0.705020 (± 7). 1968,P37(307)b: initial value, calculated from Rb 68.39 ppm, Sr 426.87 ppm; ^87^Sr/^86^Sr 0.704176 (± 7)

1. Beforsites (apatite–dolomite carbonatites) which are characteristic of early carbonatites and contain lozenge-shaped apatite, phlogopite, REE-bearing perovskite, baddeleyite and olivine
2. REE-poor carbonatites, which comprise:
  - Light-coloured ankerite/ferroan dolomite carbonatite with little REE mineralisation
  - Late, Mn-rich, REE-poor carbonatite
  - Late calcite veins
3. REE-rich carbonatites, including dark Fe- and Mn-rich carbonatite with monazite–strontianite–baryte pseudomorphs (e.g. Figure 2)
4. Monazite-rich quartz rocks from within the main intrusion
5. Quartz rocks from outside of the main intrusion
6. Fenites and country rock gneiss.

### Major and trace elements

The samples chosen for whole-rock analysis include apatite-bearing dolomite carbonatite; REE-poor dolomite carbonatites; REE-rich specimens; and coupled REE veins with their host carbonatites. The scarcity of material available precluded analysing some specimens, such as the REE-rich quartz rocks.

Samples were split from the main specimen, keeping the sample size as large as possible, crushed in a jaw crusher and ground using a TEMA mill to < 180 µm. Major and trace elements were determined by ICP-OES (ARL 3410), pyrohydrolysis and CHN analysis at the Natural History Museum, London. For major element determinations, 100 mg samples were fused with 500 mg LiBO_2_ in Pt–Au crucibles and the resulting glass beads dissolved in dilute HNO_3_. Some dark residues were dissolved by the addition of 5 ml HCl, before making to 250 ml with water. For the trace elements, including the REE, 1 g samples were treated with 10 ml 50% nitric acid and evaporated to dryness followed by digestion with 30 ml HF plus 5 ml HClO_4_ until fuming, and then re-dissolved in 25 ml 20% HNO_3_. A few drops of conc. H_2_O_2_ were added to dissolve manganese oxides before diluting to 250 ml with water. During dissolution, a precipitate formed in some samples, identified as barium sulphate by XRD. This would cause underestimation of Ba concentration, but because Sr and REE are minor components in barium sulphate, these are unlikely to be affected. No relation between total concentration and BaO content is present, but high SrO samples correspond to lower totals. This is likely due to the Sr value being in excess of the Sr standard defining the calibration range. Sulphur was determined, along with fluorine, by pyrohydrolysis-ion chromatography. Carbon dioxide and water were determined by combustion analysis using a CHN analyser.

### Carbon and oxygen isotopes

Carbon and oxygen isotope ratios were determined in a variety of mineral separates and concentrates. Concentrates were used when insoluble contaminant phases, such as monazite, baryte and quartz, were abundant. In addition, a bulk powder was prepared from three beforsite samples (SoS 168–170) using a handheld drill.

Ankerite/ferroan dolomite was concentrated by magnetic separation. Most of the carbonatites contained ankerite/ferroan dolomite of two or three different magnetic susceptibilities reflecting different Fe contents. The colour of the concentrates was noted during preparation. Strontianite and calcite were concentrated in the non-magnetic residue and separated using methyl iodide (3.31 g cm^−3^). Quartz separates were made by a combination of magnetic separation and acid dissolution. Baryte and quartz were separated by heavy liquid (bromoform, 2.82 g cm^−3^). Composite grains and goyazite–florencite contamination was removed by hand-picking, and each sample was checked using SEM–EDS to assess purity.

Most C and O data were acquired at the NERC Isotope Geosciences Laboratory (NIGL). CO_2_ was extracted offline in vacuo from approximately 10 mg of carbonate by reaction with phosphoric acid at 25.2 °C, essentially following the method of McCrea (1950). The liberated CO_2_ was cryogenically purified and collected for analysis on a 90 cm sector, triple collector, dual-inlet, mass spectrometer. The data were corrected for instrumental and isobaric effects using the methods of Craig (1957) and the results expressed as δ^13^C and δ^18^O‰ relative to VPDB and VSMOW, respectively. Overall analytical reproducibility for δ^18^O and δ^13^C was ± 0.1‰ (1*σ*). In addition, three samples (SoS 168–170) were analysed at the Environment and Sustainability Institute, University of Exeter, using a Sercon 20-22 gas-source mass spectrometer in multiflow configuration. Samples (~ 0.5 mg) were reacted with approximately 100 µl of anhydrous phosphoric acid at 70 °C. Analysis followed routines described by Spötl and Vennemann (2003). Overall analytical reproducibility for δ^18^O and δ^13^C was ± 0.03 and 0.19‰ (1*σ*), respectively.

Quartz samples were analysed at NIGL employing the oxygen liberation technique of Clayton and Mayeda (1963), but utilising ClF_3_ as a reagent instead of BrF_5_ (Borthwick and Harmon 1982). Extractions were performed at 526 ± 5 °C. The oxygen yields were converted into CO_2_ by reaction with a platinised graphite rod heated to 675 °C by an induction furnace. CO_2_ yields were measured on a capacitance manometer and CO_2_ isotopic ratios were measured on a CJS Sciences Ltd. Phoenix 390 mass spectrometer (re-built VG 903 automated triple collector machine). The δ^18^O_SMOW_ results were normalised through the NIGL laboratory quartz standard (LQS: Lochaline Glass Sand) and quoted relative to the international standard quartz NBS 28 (African Glass Sand). Determinations of the LQS standard gave a 1σ reproducibility of 0.16‰, ten replicate analyses of a range of control samples exhibited an average difference of 0.13‰.

### ^87^Sr/^86^Sr isotopes

Strontium isotope analyses were performed at NIGL. Mineral separates, of approximately 150–400 mg, were used for most samples. Whole-rock samples and mineral concentrates were used for samples outside of the main intrusion because these specimens only contain one significant Sr mineral which dominates the isotopic signature. Dissolution techniques varied according to the sample material, H_2_SO_4_ was used to dissolve monazite, HCl for apatite and florencite, HF for the fenites and HF followed by HNO_3_ for quartz–fluorite rocks. After dissolution, the samples were evaporated and converted into chloride by the addition of HCl. Rb and Sr were separated using conventional ion-exchange columns and ratios were determined using TIMS. The ^86^Sr/^88^Sr normalisation factor used for Sr was 0.1194 (Nier 1938; Steiger and Jäger 1977). Over the period of data acquisition, 89 analyses of NIST SRM 987 gave an average value of 0.710194, with 2 standard errors of the mean of 0.000004 and 2 standard deviations of 0.000037. Owing to low Rb concentrations, relative to Sr, in the carbonatites, radiogenic ingrowth is negligible and, thus, ^87^Sr/^86^Sr is representative of initial values. Only samples from fenite contain sufficient Rb such that the ^87^Sr/^86^Sr needs to be corrected.

## Results

### Major and trace elements

**Fig. 3:**
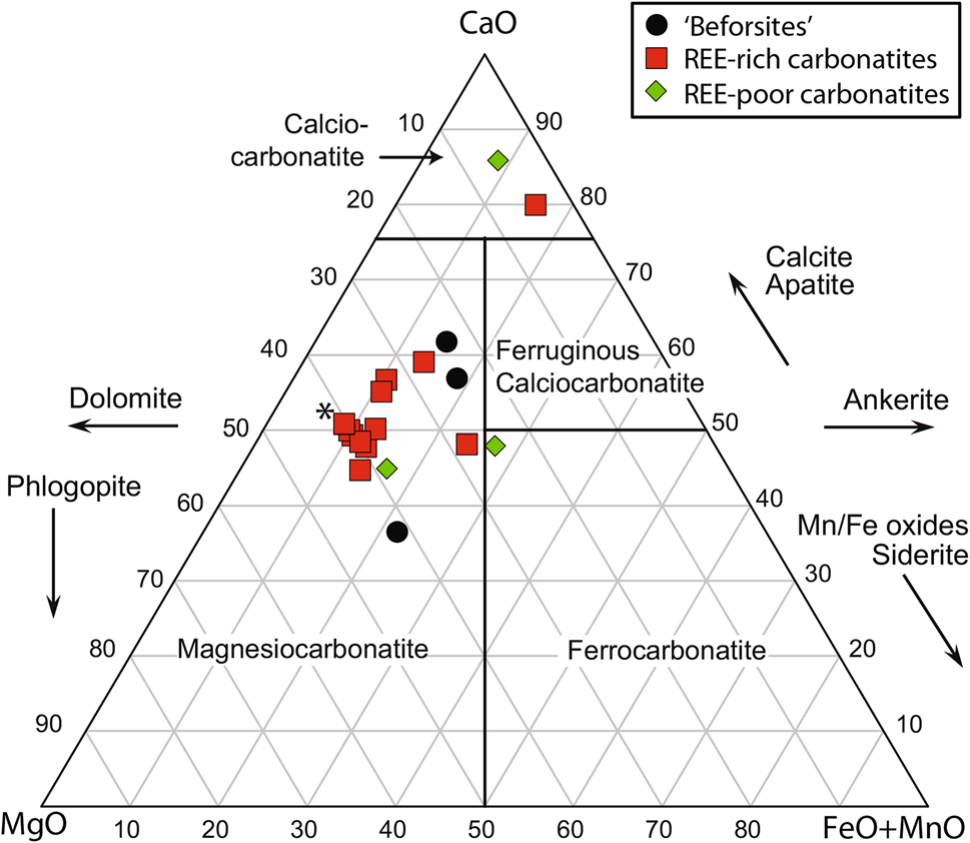
Kangankunde carbonatites plotted using molar proportions utilising the method and carbonatite nomenclature of Gittins and Harmer (1997). Arrows denote the principal mineralogical controls on the whole-rock composition. Starred data-point from Verplanck et al. (2016)

**Fig. 4:**
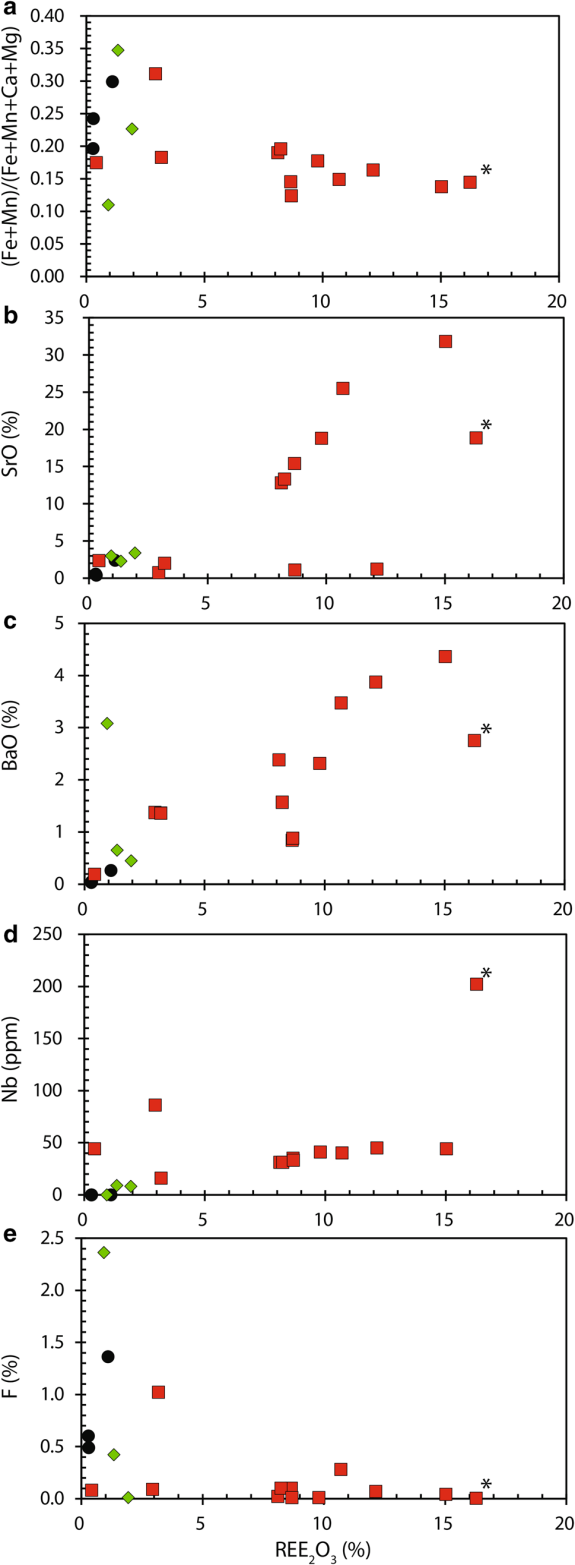
Selected plots of major and minor elements in Kangankunde carbonatites. Symbols same as in Fig. 3. Starred data from Verplanck et al. (2016)

Major and trace element data are presented in Supplementary Table 2 which includes a single analysis from Verplanck et al. (2016). Using the revised carbonatite classification diagram of Gittins and Harmer (1997), most samples plot in the magnesiocarbonatite field, close to the line defining the trend from dolomite to ankerite (Fig. 3). Some samples are more magnesium rich, due to higher modal abundances of phlogopite, while slightly Ca-rich samples have higher apatite concentrations. Two samples plot in the calciocarbonatite field but these are dolomite carbonatites which contain secondary calcite. There is no apparent relationship between the degree of Fe enrichment and the REE_2_O_3_ content. The highest ratios of Fe + Mn to Mn + Ca occur in the beforsites and REE-poor rocks (Fig. 4a).

The REE contents of the carbonatites range from 0.25 wt% REE_2_O_3_ in beforsite up to 8–15 wt% in the REE-rich carbonatites. There is a clear division between the REE-rich samples and the other rocks, including rocks very closely related to REE-rich carbonatites [e.g. BM 1993, P4 (5)]. This is especially illustrated by the difference between the REE contents of the mineralised veins and the less-mineralised host carbonatites. Two of the beforsites [BM 1962, 72 (50) and (58)] have the lowest REE concentration but a third [BM 1962, 72 (59)] is slightly more REE-enriched owing to incipient REE mineralisation (Wall and Mariano 1996). Higher REE_2_O_3_ contents correspond to elevated Sr and Ba concentrations (Fig. 4b, c). This reflects the association between monazite, strontianite and baryte in the REE mineral assemblage. Niobium also is associated with increasing REE contents and, in the REE-rich rocks, Nb is hosted in accessory amounts of Sr-bearing pyrochlore (Fig. 4d). This is somewhat unusual because pyrochlore, the principal host for Nb in carbonatites, generally occurs in calcite carbonatites (Chakhmouradian 2006). There is little relationship between the REE and F at Kangankunde (Fig. 4e). This reflects the low abundance of REE–fluorcarbonates relative to monazite-(Ce). Higher F contents occur in the phlogopite- and apatite-rich beforsite samples.

**Fig. 5:**
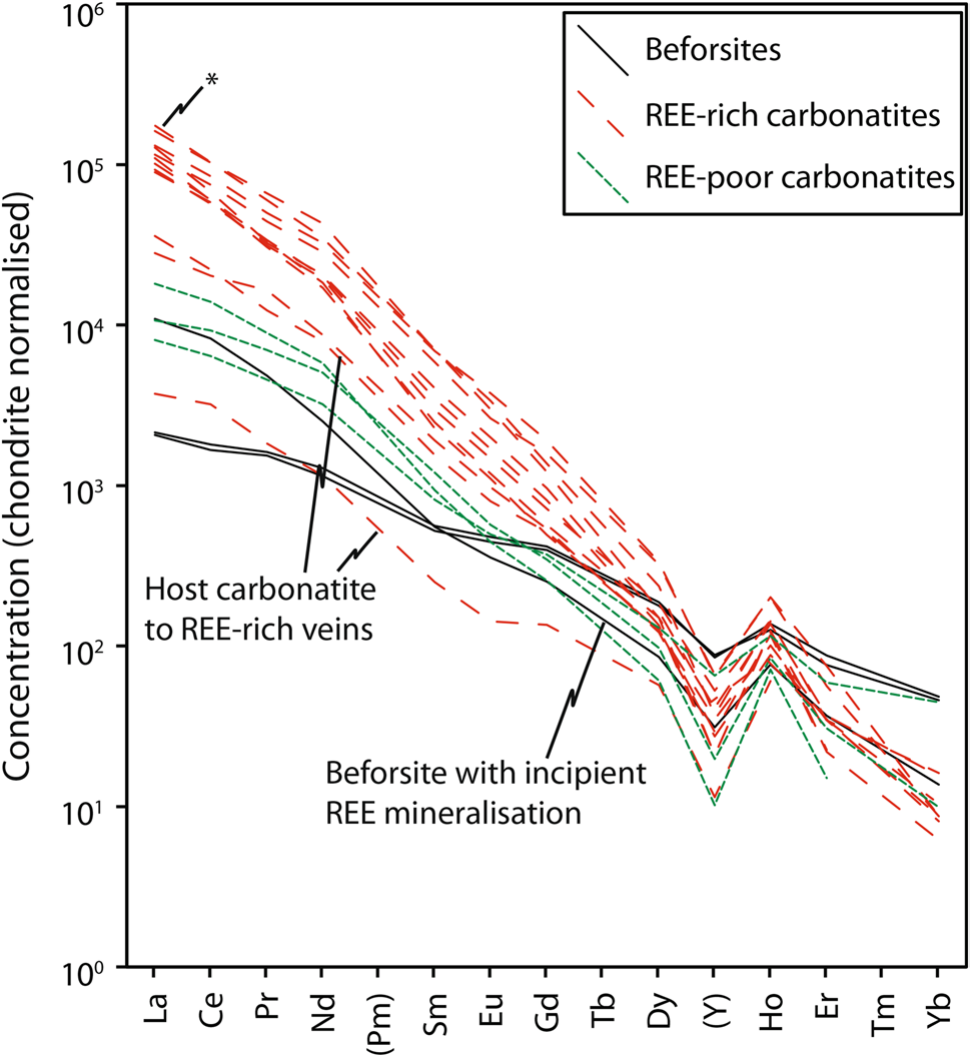
Chondrite-normalised REE concentrations of Kangankunde carbonatite whole rock analyses; starred data from Verplanck et al. (2016). Normalisation values from McDonough and Sun (1995)

All of the Kangankunde rocks are highly enriched in the light (L)REE (Fig. 5). Beforsite samples have the flattest REE patterns, except for the sample with incipient REE mineralisation which has slightly elevated LREE contents [BM 1962, 72 (59); Wall and Mariano (1996)]. REE-rich samples have the steepest REE distributions, except where the host carbonate has been separated from REE veins. These plot in a similar range to the REE-poor carbonatites. All of the analysed samples display a prominent negative Y anomaly, which is most pronounced in the more LREE-rich samples.

### Carbon and oxygen in carbonates

C and O isotope data from carbonates are considered relative to the Primary Igneous Carbonatite (PIC) box. This box represents the compositional range of globally distributed carbonatites, unaffected by surficial processes, and is considered representative of the mantle source composition (Taylor et al. 1967; Deines 1989; Keller and Hoefs 1995; Demény et al. 2004; Jones et al. 2013). In this contribution we use the PIC field of Demény et al. (2004). Overall, there is a wide range of δ^13^C and δ^18^O values in the carbonate minerals at Kangankunde (Table 1). However, the different carbonate minerals plot into distinct groups, summarised in the following four paragraphs.

**Fig. 6:**
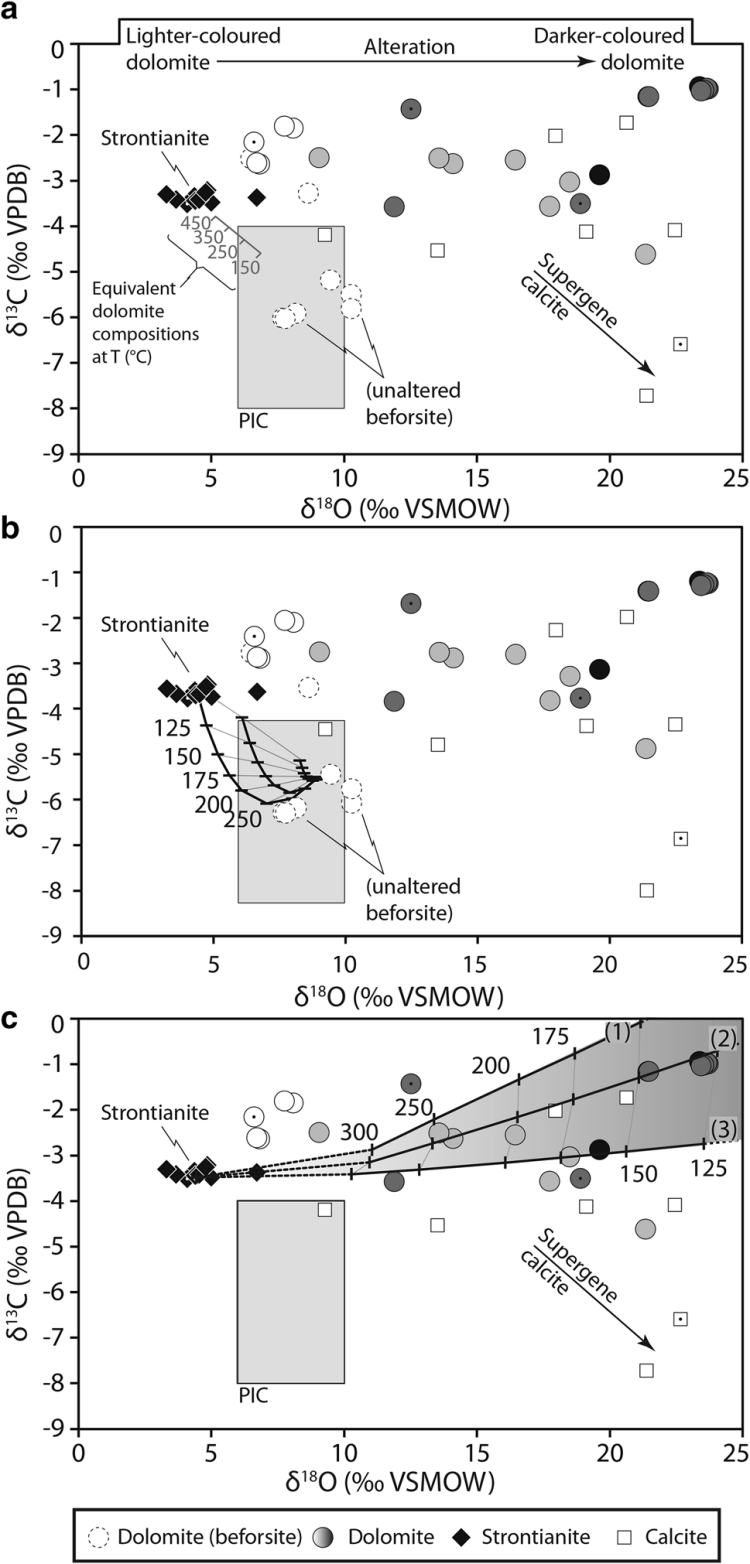
Oxygen and carbon isotope ratios from Kangankunde carbonates. PIC = primary igneous carbonatite box from Demény et al. (2004). All data-points represent REE-rich carbonatites with the exception of dashed circles (beforsite) and dotted data-points (REE-poor carbonatites). **a** Strontianite (diamonds) plots in a distinctive group and the equivalent dolomite compositions for a range of temperatures are indicated for comparison. Ferroan dolomite (circles) span a range of δ^18^O values, corresponding to the darkness of the mineral grain analysed, as represented by the infill colour. No colour data are available for dolomite represented by dashed circles (beforsite). Calcite (squares) broadly follows the dolomite trend, but distinctly late supergene calcite is progressively ^13^C-depleted. **b** The same data compared with model carbonate compositions forming from a CO_2_-rich fluid, representative of a degassing PIC source at a range of temperatures. **c** Same as **b**, but with modelled carbonate compositions from a H_2_O rich fluid, at different degrees of alteration and temperature. Path (1) represents complete replacement at 100 °C, while (2) and (3) represent 50 and 10% replacement at 100 °C, respectively

Only analyses of ferroan dolomite from the beforsite (apatite–dolomite carbonatite) plot in the PIC box (Fig. 6a). A beforsite containing incipient REE mineralisation (BM 1962, 73 (59) [Wall and Mariano, 1996]) has higher δ^13^C values, similar in composition to light-coloured carbonates from the mineralised samples, and plots outside of the PIC box.

Ferroan dolomite/ankerite from the REE-rich carbonatites spans a wide range of δ^18^O values, extending from approximately + 6 to + 24‰. The corresponding δ^13^C ranges, however, are more restricted, ranging from − 5 to − 1‰, outside of the PIC box (Fig. 6a). Oxygen and, to a lesser extent, carbon isotope ratios in the ferroan dolomite/ankerite vary in relation to the ‘darkness’ of the carbonate analysed. While this observation is qualitative, it appears that the darker the carbonate, the more positive the δ^18^O value (Fig. 6).

In contrast to the ferroan dolomite, strontianite from all rocks plots in a tight cluster of + 3 to + 5‰ δ^18^O and − 3.5 to − 3.2‰ δ^13^C. While relatively close to the values for the light-coloured ferroan dolomite samples (0.6–2.8‰ lower in δ^18^O), these two phases are unlikely to have crystallised in equilibrium: this is supported by the pseudomorph textures, which indicate later crystallisation of strontianite through replacement of a hexagonal phase (e.g. Fig. 2), and through comparison with C and O fractionation factors between dolomite and strontianite at different temperatures. For example, using the O-isotope fractionation factors of Horita (2014) (80–350 °C) and Chacko and Deines (2008) (350–500 + °C), these two minerals should be in equilibrium between approximately 250 and 400 °C. However, using the C-isotope fractionation factors of Deines (2004), these two phases cannot be in equilibrium as fractionation between dolomite and strontianite should result in strontianite having more positive δ^13^C values. Nevertheless, it is possible that, across the suggested temperature range, the strontianite is in equilibrium with carbonate of a composition equivalent to the top left corner of the PIC box (Fig. 6a).

Data from calcite broadly follow the same trend as the dolomite, but a calcite vein from a joint plane [BM, 1993, P4 (18)], has high δ^18^O and relatively low δ^13^C values, suggesting a low temperature of formation and the incorporation of some organic-derived C. This calcite is, therefore, thought to be deposited from surface-derived waters, probably significantly later than the other carbonates at Kangankunde. Calcite separated from within one rock [BM, 1993, P4 (9)] plots in the same field and is thought to be of the same origin. There is no evidence for primary calcite at Kangankunde.

### Oxygen in quartz

Oxygen isotope values in quartz range from + 8.3 to + 17.7‰ (δ^18^O vs SMOW; Table 1). However, the lowest values are restricted to low-grade fenite samples, and most mineralised specimens plot between + 12.5 and + 17.7‰. In sample BM, 1962, 73 (100), where data are available for quartz (+ 13.2‰ δ^18^O) and carbonates (+ 6.7 to + 9.3‰ δ^18^O), these minerals are unlikely to be in equilibrium. Comparison between dolomite–H_2_O (Horita 2014) and quartz–H_2_O (Matsuhisa et al. 1979; Zhang et al. 1989) fractionation factors, at temperatures of 250–400 °C, indicates that Δ^18^O_Dol-Qrtz_ should not exceed 1.1‰: far lower than the observed difference.

### ^87^Sr/^86^Sr isotopes

**Fig. 7:**
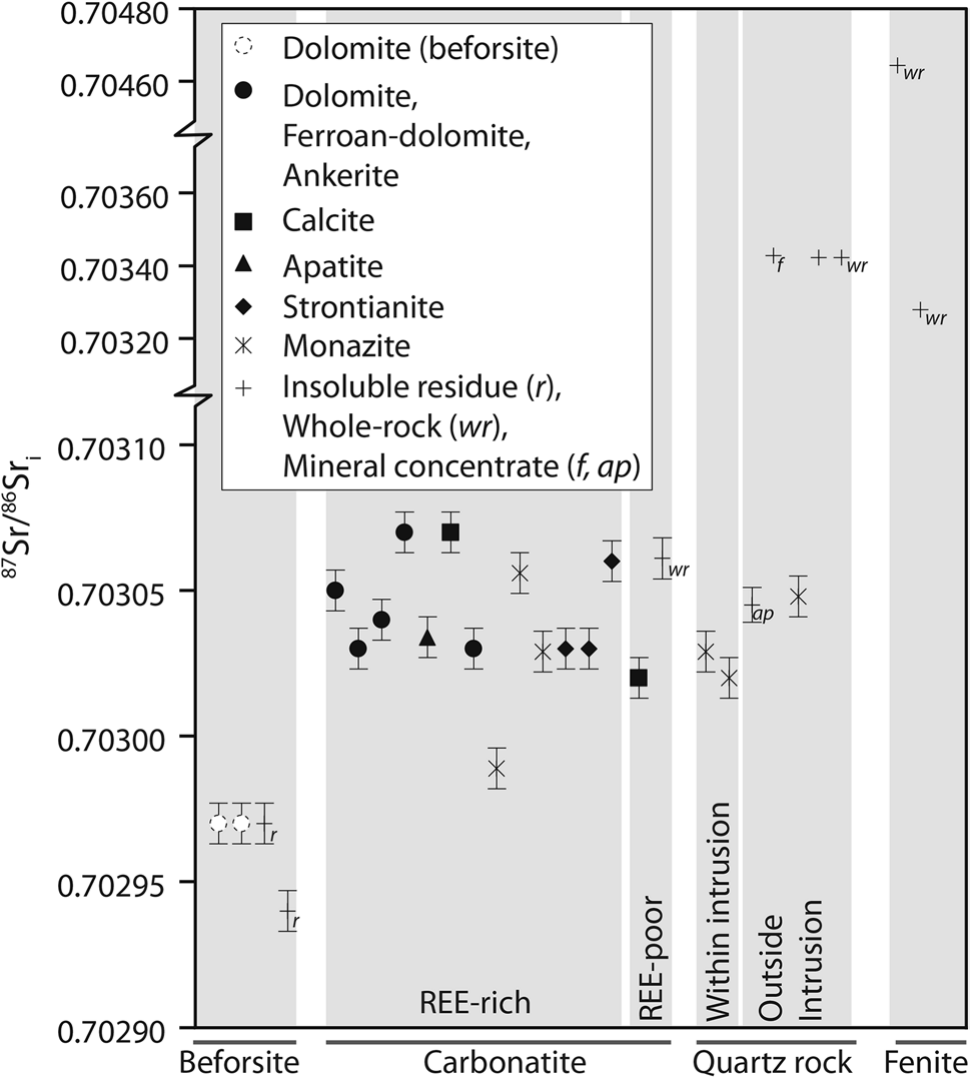
^87^Sr/^86^Sr ratios of minerals from different rock types at Kangankunde. Note the similar beforsite values, which are markedly lower than the other carbonatite analyses at Kangankunde. Many quartz rocks have the same ^87^Sr/^86^Sr ratios as carbonatites, with the exception of whole-rock samples and fluorite concentrate, which occur outside of the main intrusion and are likely contaminated with fenite/country rock. *Ap* apatite, *f* fluorite

^87^Sr/^86^Sr isotope ratios for almost all samples in this study lay in a narrow band between 0.70302 and 0.70307 (*n* = 20), similar to the range of results of Ziegler (1992), affirming a common carbonatite-derived origin for all samples (Table 1; Fig. 7). The beforsite samples all have slightly lower ratios, falling within a narrow envelope of 0.70296–0.70298 (*n* = 4). Other exceptions include fenite samples and the quartz–fluorite and quartz–florencite rocks. Low-grade quartz fenite has an initial ratio (see Table 1 for Rb concentrations and corrections) of 0.70464, while a fenite vein within the same rock has a ratio of 0.70328. The quartz–fluorite and quartz–florencite rocks are the furthest two samples outside of the main carbonatite. These specimens have elevated ^87^Sr/^86^Sr ratios, suggesting some isotopic mixing with a country rock source. Excluding two data-points from calcite, there is a correlation between δ^13^C and ^87^Sr/^86^Sr (*R* ^2^ = 0.5794). However, this correlation is predominantly controlled by low ^87^Sr/^86^Sr ratio of two beforsite data-points and, therefore, is probably not significant. There appears to be little relation between ^87^Sr/^86^Sr and δ^18^O values.

## Discussion

### Relationship between beforsite and the mineralised carbonatites

The earliest carbonatite at Kangankunde is beforsite (apatite–dolomite carbonatite), which forms a small central plug (Fig. 1). Based on the abundance of phlogopite in this rock, Garson and Campbell Smith (1965) suggested that it is a ‘carbonatised nephelinite’ where the carbonate is an alteration product related to later REE-bearing carbonatites. However, the new C and O isotope data plot within the PIC field and indicate that this rock type is a primary magmatic carbonatite. This interpretation is supported by the presence of ovoid apatite grains, which are commonly an early liquidus phase in carbonatites (Le Bas 1989), as well as evidence of REE-rich fluids reacting with apatite and forming incipient REE mineralisation (Wall and Mariano 1996).

Beforsite is unlikely to have any parental relationship to the REE-mineralised carbonatites at Kangankunde. It is mineralogically distinct, with much ovoid apatite, altered olivine and accessory baddeleyite and perovskite (Garson and Campbell Smith 1965; Wall and Mariano 1996). Its ^87^Sr/^86^Sr ratios also form a separate population (Fig. 7). There is no clear compositional relationship between beforsite and REE-rich carbonatite, and beforsite has distinctly lower C isotope ratios while retaining similar O isotope values. It is, therefore, difficult to envisage any fractionation or hydrothermal process which relates these rock types as both would increase the O and C isotope ratios (e.g. Deines 1970, 1989; Santos and Clayton 1995; Ray and Ramesh 1999, 2000; Trofanenko et al. 2016). This interpretation is supported by the size of the two carbonatite bodies exposed; the earlier apatite-bearing dolomite carbonatite being much smaller than the later REE-rich carbonatites (Fig. 1).

### Order of events and number of fluid generations

The exact number of generations of fluid involved in the mineralising process at Kangankunde, is somewhat equivocal. This is a common problem in REE-mineralised carbonatites, as they are highly susceptible to auto-metasomatism, dissolution, and alteration by groundwater. However, combining the field and mineralogical evidence outlined in previous contributions (e.g. Garson and Campbell Smith 1965; Wall and Mariano 1996; Duraswami and Shaikh 2014; Broom-Fendley et al. 2016a) with the new isotope data presented herein, it is possible to outline the order of events and postulate two major fluid generations. These different fluid generations are divided into (1) mineralising fluids, responsible for altering burbankite to the strontianite–baryte–monazite mineral assemblage; and (2) subsequent altering fluids, which modify the isotopic composition of the host carbonates and can erroneously be associated with the mineralising event. These are discussed in the following sub-sections. This separation of fluids is arbitrary and is intended to represent processes at different stages of the evolution of the deposit, rather than separate fluids from different sources.

#### Source of REE mineralising fluid(s) and nature of the mineralisation

The negative Y anomaly in all of the REE-rich samples is compelling evidence for interaction between the REE and a hydrothermal fluid (c.f. Bau 1996). The nature of the ligands involved in this fluid, however, is less clear and there is little direct evidence to help with this. In fluids capable of transporting the REE, fluoride complexation has been shown to preferentially transport Y over the HREE (Bau and Dulski 1995; Loges et al. 2013), and Bühn (2008) suggested that the negative Y anomaly in carbonatites was evidence for fluid transport of Y and the HREE away from carbonatites. However, the F-poor nature of most of the samples at Kangankunde suggests a diminished role for REE–fluoride complexes. Furthermore, carbonate complexes are unlikely because they would cause Y to behave more like the LREE and thus should create a positive Y anomaly (Bau and Dulski 1995). Chloride is currently commonly favoured as the most important ligand for transporting the REE (e.g. Williams-Jones et al. 2012; Downes et al. 2014; Trofanenko et al. 2016; Broom-Fendley et al. 2016a, 2017a) but there is not yet any fluid inclusion evidence at Kangankunde to support this. Given the circumstantial nature of the evidence for different fluid compositions, it is difficult to conclude how the REE are transported at Kangankunde. However, the low F contents from the whole-rock data, in combination with the results of recent experimental work, summarised above, strongly suggest that F has a diminished role for REE complexation and deposition.

There is little evidence for any contribution from external fluids for the REE mineralisation process. ^87^Sr/^86^Sr values are consistent in the REE-rich carbonatites (Fig. 7), although this is, in part, an effect of their very high Sr contents making ^87^Sr/^86^Sr an insensitive parameter. The tight clustering of strontianite δ^18^O and δ^13^C values, representing the REE mineral assemblage, can be achieved through two mechanisms: (1) equilibrium fractionation between strontianite and dolomite in a carbonatite-derived fluid/melt or (2) equilibrium degassing from a PIC source. Equilibrium fractionation, at a range of temperatures, between strontianite and a PIC fluid is outlined in Fig. 6a. This demonstrates that strontianite is in equilibrium with primary igneous carbonatite at a temperature range of approximately 350 °C or less (Fig. 6a; Chacko and Deines 2008; Horita 2014). The role of degassing can be evaluated using a model whereby changes in C and O isotope ratios between carbonate (taking calcite as a proxy for dolomite), H_2_O, and CO_2_ are defined as a function of two mass-balance equations (Santos and Clayton 1995; Ray and Ramesh 1999):

1 $$\updelta^{13} {\text{C}}_{\text{rock}}^{\text{final}} = \frac{{\left( {\frac{{F_{\text{C}} }}{{R_{\text{C}} }}} \right)(\updelta^{13} {\text{C}}_{\text{fluid}}^{\text{initial}} + \Delta_{{{\text{rock}} - {\text{fluid}}}}^{\text{C}} ) +\updelta^{13} {\text{C}}_{\text{rock}}^{\text{initial}} }}{{1 + \left( {\frac{{F_{\text{C}} }}{{R_{\text{C}} }}} \right)}},$$

and

2 $$\updelta^{18} {\text{O}}_{\text{rock}}^{\text{final}} = \frac{{\left( {\frac{2r + 2}{3r}} \right)\left( {\frac{{F_{\text{C}} }}{{R_{\text{C}} }}} \right)(\updelta^{18} {\text{O}}_{\text{fluid}}^{\text{initial}} + \Delta_{{{\text{rock}} - {\text{fluid}}}}^{\text{O}} ) +\updelta^{18} {\text{O}}_{\text{rock}}^{\text{initial}} }}{{1 + \left( {\frac{2r + 1}{3r}} \right)\left( {\frac{{F_{\text{C}} }}{{R_{\text{C}} }}} \right)}},$$

where *F*_C_ = moles of carbon in the fluid, *R*_C_ = moles of carbon in the rock, ∆_rock–fluid_^C^ = difference in carbon isotopes between the rock and the fluid, *r* = molar ratio of CO_2_ to H_2_O in the fluid (here considered as 1000, to reflect a CO_2_-rich fluid), and:

3 $$\Delta_{{{\text{rock}} - {\text{fluid}}}}^{\text{O}} = 10^{3} \ln\upalpha^{18} {\text{O}}_{{{\text{cc}} - {\text{CO}}_{2} }} + 10^{3} \ln (1 + 2r) - 10^{3} \ln (2r +\upalpha^{18} {\text{O}}_{{{\text{H}}_{2} {\text{O}} - {\text{CO}}_{2} }} ),$$

where $\upalpha^{18} {\text{O}}_{{{\text{cc}}{-}{\text{CO}}_{2} }}$ and $\upalpha^{18} {\text{O}}_{{{\text{H}}_{2} {\text{O}} - {\text{CO}}_{2} }}$ are fractionation factors between calcite–CO_2_ and H_2_O–CO_2_, at a given temperature. Calcite is assumed to represent carbonatite and fractionation factors for calcite–CO_2_ are taken from Chacko et al. (1991), while H_2_O–CO_2_ is taken from Richet et al. (1977). Initial δ^18^O and δ^13^C values used are + 4.5‰ and − 3.5‰, similar to those of strontianite. Model calculations are included in the supplementary material.

The modelled effects of different degrees of degassing are presented in Fig. 6b. In these models, the degree of exchange between the CO_2_-rich fluid and the rock (*F*_C_/*R*_C_) is related to temperature: as temperature decreases the degree of exchange increases. The modelled temperature range is between 350 and 100 °C, and the corresponding *F*_C_/*R*_C_ ranges are from (1) 0–1, representing complete exchange, (2) 0–0.5, and (3) 0–0.1, representing partial exchange between the fluid and the crystallising carbonate (Fig. 6b).

As can be seen in Fig. 6b, degassing represents an equally viable mechanism (to fractionation) for obtaining the elevated strontianite δ^13^C and decreased δ^18^O ratios relative to a hypothetical PIC source. This similarity is because, in terms of their effect on the carbonate stable isotope composition, both mechanisms are variations on carbonate–CO_2_ fractionation. The process of degassing, however, typically results in brecciation of the host rock. While breccia pipes and degassing structures are apparent in many carbonatites (e.g. Songwe Hill and Chilwa Island, Malawi; Broom-Fendley et al. 2017b, Garson and Campbell Smith 1958), they are not widespread at Kangankunde. Thus, fractionation between strontianite and a PIC fluid (Fig. 6a) is favoured over degassing as the isotopic source of the REE mineralising fluids.

Unfortunately, there is no burbankite at Kangankunde with which to compare the strontianite values, so it is difficult to infer compositional changes during pseudomorph formation. Strontianite C and O isotope compositions are lower than those from burbankite at other localities (e.g. δ^18^O, + 7.3 to + 7.6‰, Khibiny; + 8.1‰, Vuoriyarvi; + 9.7‰, Bear Lodge; Zaitsev et al. 2002; Moore et al. 2015), but these are all close to, or within, the PIC box.

The similar composition of strontianite to primary magmatic carbonatites indicates that alteration and breakdown of burbankite to the monazite-bearing pseudomorph assemblage is likely to have occurred soon after its formation. It is, therefore, suggested that burbankite crystallisation and breakdown occurs over a narrow temperature window. Interaction with a cooling deuteric fluid is the most likely cause of this breakdown, imparting a negative Y anomaly. However, the REE would rapidly reprecipitate owing to the low solubility of monazite-(Ce) (Poitrasson et al. 2004; Cetiner et al. 2005; Louvel et al. 2015; Zhou et al. 2016). The C and O data for strontianite, in equilibrium with the monazite-(Ce), indicate that no meteoric water input is required for this process.

The formation of burbankite probably occurred in a transition environment, from magmatic to (carbo)-hydrothermal fluids, as indicated by the pegmatitic nature of the pseudomorphs (Wall 2000; Wall et al. 2001) and unlikely to be related to late-stage hydrothermal fluid activity as proposed at other complexes by Zaitsev et al. (2002). The strontianite stable isotope data presented here contrast with analyses of the pseudomorph assemblage from Khibiny and Vuoriyarvi which have much more ^18^O-enriched compositions, up to 18‰ (Zaitsev et al. 2002). A possible reason for this is discussed below.

#### Alteration after the formation of the main mineral assemblage

The dominant trend in the C and O data, towards higher δ^18^O values (Fig. 6a), is probably the result of increasing degrees of exchange between dolomite and later, cooling, deuteric fluid(s) (e.g. Demény et al. 2004). The role of this alteration can be evaluated using the models outlined in Eqs. 1–3, changing *r* to 0.001 to reflect a low CO_2_ fluid (see supplementary information and Fig. 6c). In these models, the effect of differing degrees of exchange (equivalent to degree of alteration) is also related to temperature: as temperature decreases the degree of alteration increases. Increasing the degree of exhange while lowering fluid temperature is justified as carbonate solubility increases as temperature decreases (Dolejš and Manning 2010). Support is also lent through the increasing ‘darkness’ of the dolomite samples as δ^18^O increases. This colour change is likely to be due to the breakdown of ferroan dolomite, which is less stable than its magnesian counterpart (Chai and Navrotsky 1996), into iron oxides and dolomite or, occasionally, calcite. Isotopic exchange with water causes in-situ alteration of the ferroan dolomite resulting in Fe-oxide exsolution and thus a darker mineral grain. Textural evidence at Kangankunde, such as etching along cleavage and spongey, pitted surfaces on the dolomite, supports this interpretation (Duraswami and Shaikh 2014). The dolomite data broadly match the trend of the models, with the darkest samples generally having higher δ^18^O values (Fig. 6c). Combined, the models and data indicate that carbonate dissolution and isotopic exchange, as temperature decreases, are likely to be the main controls on the dolomite composition. Calcite compositions, with the exception of those from supergene veins, broadly follow the main dolomite trend, indicating that it is affected by a similar process. Strontianite does not appear to be affected by late re-equilibration, suggesting that it is less susceptible to isotopic exchange than dolomite or calcite. The reason for this is unknown, and beyond the scope of this manuscript.

The above processes illustrate the cooling of a single fluid but this does not preclude the possibility that multiple phases of re-circulating fluid are present at Kangankunde. Multiple fluid generations, if they are of the same composition, would likely have the same effect on the dolomite C and O isotope values as a single cooling fluid. The modelled processes could explain the difference between the stable isotope composition of the pseudomorph assemblage at Khibiny and Vuoriyarvi and the strontianite at Kangankunde. The analyses of this assemblage at Khibiny and Vuoriyarvi included calcite, which may have been susceptible to subsequent isotopic exchange. Furthermore, the above process does not eliminate the possibility that the REE were re-distributed by these cooling, altering fluids. Such a process is indeed likely at many carbonatites and is supported by the presence of REE minerals in veins and vugs (e.g. Doroshkevich et al. 2009; Downes et al. 2014; Broom-Fendley et al. 2016b). However, the model results and dolomite data illustrate the difficulty in unequivocally tying carbonate isotopic compositions with REE mineralisation. To obtain representative stable isotope data linked to the mineralisation process, it may be better to analyse the REE-bearing mineral (e.g. Broom-Fendley et al. 2016b).

### Interaction between the carbonatite and surrounding country rock

**Fig. 8:**
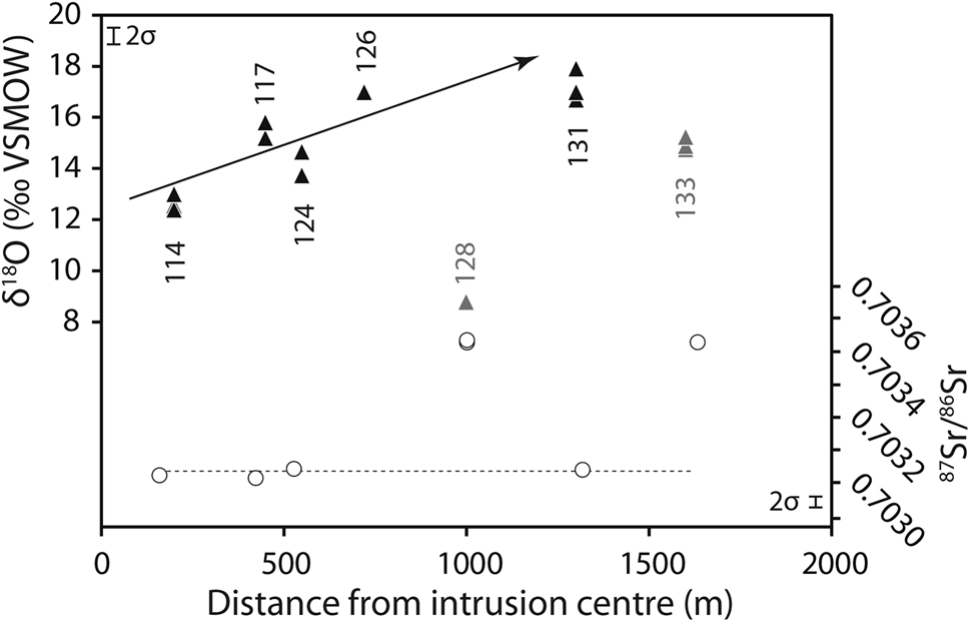
Quartz oxygen isotope values (triangles) and ^87^Sr/^86^Sr ratios (circles) in quartz rocks from within and outside of the main intrusion. Oxygen isotope values increase with greater distance from the main intrusion, excluding rocks which have incorporated a country rock component based on elevated ^87^Sr (greyed out: 128, 133). Three-digit labels reflect BM sample numbers. Distances are approximate. Analytical uncertainty is included next to the axes

Both within and around Kangankunde, unusually quartz-rich rocks occur which are commonly REE-mineralised (Garson and Campbell Smith 1965; Wall 2000; Broom-Fendley et al. 2016a). These are found up to 1.6 km away from the main intrusion (Fig. 1). The consistent ^87^Sr/^86^Sr ratios (within uncertainty) indicate that, with the exception of the quartz–fluorite and quartz–florencite rocks [BM 1962, 73 (128) and (133)], the quartz rocks are likely to be derived from the carbonatite (Fig. 8). The two excluded samples, with higher ^87^Sr/^86^Sr ratios, are likely to be contaminated with a small amount of country rock. The sample scarcity of the quartz rocks means no whole-rock data are available to model mixing between the carbonatite and country rock end-members. However, fenite samples are broadly representative of the country rock and these have a significantly more radiogenic ^87^Sr/^86^Sr ratio than the carbonatite (Table 1).

Rare earth mineralisation in the quartz-rich rocks suggests carbonatite-derived fluids have the capability to transport the REE well outside of the main intrusion and into the fenite aureole. A comparison of the distance of these rocks from the main carbonatite centre (taken as the middle of the REE-rich carbonatite) with their quartz O isotope composition illustrates that more distal samples have higher δ^18^O values than their closer counterparts (Fig. 8). Deines (1970) noted a similar trend in non-REE-mineralised calcite in ijolite at the Oka complex, Canada, where the ^18^O content increased away from the carbonatite contact. The simplest interpretation of these examples is that they are caused by quartz (or calcite) crystallising at lower temperatures away from the main carbonatite intrusion. However, petrographic studies of the Kangankunde rocks indicate that quartz formed after the REE mineralisation (Wall and Mariano 1996; Broom-Fendley et al. 2016a), and so its composition does not provide information about the conditions of REE transportation. A role of meteoric water in these more distal samples cannot, therefore, be excluded.

## Conclusions

The main conclusions from this work are as follows:

1. Strontianite is in isotopic equilibrium (δ^18^O and δ^13^C) with a hypothetical PIC-derived fluid, suggesting that the monazite-(Ce), strontianite, baryte pseudomorph assemblage formed soon after the probable original burbankite crystals. Given the similar ranges of δ^18^O and δ^13^C values with PIC, and consistent ^87^Sr/^86^Sr values for all the REE-rich carbonatites, no significant input of meteoric water was required for this process, which is the result of deuteric (carbo)-hydrothermal fluid.
2. Dolomite and calcite, in the REE-rich samples, have high δ^18^O values relative to strontianite and the PIC field. Modelled fluid compositions suggest these values are caused by increasing degrees of alteration by low-temperature fluids. Such models are corroborated by the increasing ‘darkness’ of dolomite caused by Fe exsolution at lower temperatures. This process for formation of dark ‘oxidised’ carbonates is separate from the REE mineralisation. Use of δ^18^O and δ^13^C isotopes of calcite and dolomite in REE-rich rocks to infer fluid sources and temperatures may yield misleading results.
3. REE mineralisation in quartz rocks occurs up to 1.5 km outside of the main carbonatite and has similar δ^18^O and ^87^Sr/^86^Sr values to the REE-rich carbonatites. This similarity suggests carbonatite-derived fluids have the capability to transport the REE well outside of the main intrusion and into the fenite aureole.
4. There are two main varieties of carbonatite present at Kangankunde: small intrusions of earlier apatite-bearing dolomite carbonatite (beforsite) and later REE-rich ferroan dolomite carbonatites. These have different ^87^Sr/^86^Sr and C isotope ratios and cannot readily be linked by a common genetic process. The earlier dolomite plots into the PIC field, and is not a product of alteration of a silicate intrusion as previously proposed.

With these conclusions, we are able to deduce that the source for REE-bearing fluids is predominantly carbonatite-derived and that the majority of mineralisation occurs over a narrow temperature window. This is likely to be between 250 and 400 °C, based on equilibrium fractionation between strontianite and a hypothetical magmatic carbonatite. The fluid composition, in terms of what is complexing and transporting the REE, remains enigmatic, but we infer a weak role for fluorine.

## Electronic supplementary material

Below is the link to the electronic supplementary material.

Supplementary material 1 (DOCX 1802 kb)

Supplementary material 2 (XLSX 25 kb)
