# Supplementary material for: Deducing the source and composition of rare earth mineralising fluids in carbonatites: insights from isotopic (C, O, 87Sr/86Sr) data from Kangankunde, Malawi
Source: Contrib Mineral Petrol. 2017 Nov 9;172(11):96. doi: 10.1007/s00410-017-1412-7 (PMC6959380; doi:10.1007/s00410-017-1412-7)
Supplement: Supplementary file 1 — Supplementary material 1 (DOCX 1802 kb) [file 410_2017_1412_MOESM1_ESM.docx]

# Supplementary information

**Supplementary table 1.** Petrographic descriptions of samples analysed in this study

| **BM number** | **Field number*** | **Description** |
| --- | --- | --- |
| 1993,P4(1) | K1a | Monazite-strontianite-rich area in ankerite carbonatite. Some vein-like and disseminated monazite in ankerite. Vein is clear on the back of the specimen |
| 1993,P4(5) | K3 | Light-coloured, medium grained, carbonatite with sphalerite and a small amount of green monazite. Occasional spherulitic aggregates of monazite |
| 1993,P4(7) | K5 | Monazite-strontianite pseudomorphs in dark carbonatite with carbonate crystals up to 4mm diameter. There are some cavities in the pseudomorphs. Evidence of secondary crystallisation in the dark carbonatite because there are lighter rings of secondary carbonate. Pseudomorphs are edged with monazite and have radiating habit although the exact points of origin are difficult to determine. There are no perfect hexagonal cross sections |
| 1993,P4(9) | K7 | Dark carbonate with many poorly-formed pseudomorphs. Monazite is particularly bright-green and is associated with baryte(?) in the pseudomorphs. There are many cavities in the monazite-rich areas. Strontianite and monazite are both coarse-grained. There is radiate monazite and turbid apatite neighbouring monazite. Radiate Fe-oxides have nucleated on monazite. |
| 1993,P4(14) | K11b | Dark, rather messy carbonatite, cut by a 5 mm wide vein of white calcite on one side of the rock. There are also 1 mm wide parallel veinlets of calcite in the wall-rock. The carbonatite, which is medium-fine grained (0.5 mm) contains cavities and cross-cutting textures of pinkish carbonate. Contains some monazite in patches and veinlets. |
| 1993,P4(15) | K12 | Dark coarse-grained (2–3 mm) carbonatite with good monazite-strontianite pseudomorphs. The host carbonatite is rich in Fe oxides. Longitudinal sections of the pseudomorphs are up to 40 mm long. Cross sections have 6-sided forms but are not perfect hexagons. There are no cavities in the pseudomorphs. The pink areas at the edges of the pseudomorphs may be florencite. Veinlets of dark carbonate, 0.5 mm wide, cut the long sections of some pseudomorphs. Some pseudomorphs are mantled by a less lustrous dark carbonate. |
| 1993,P4(18) | K15 | Light-brown carbonatite, rich in green monazite and strontianite. Some evidence of pseudomorphs, although much is coarse-grained and massive. Pinkish areas could be florencite. 5mm brown carbonate vein cuts monazite |
| 1993,P4(19) | K16 | Mid-brown mottled carbonatite with crystals 1–­2 mm diameter cut by a distinct 17 mm wide monazite-strontianite vein. The vein itself is cut by <0.5 mm dark veinlets which run irregularly through the rock. In the vein, monazite is concentrated along the centre and at the margins. The carbonatite itself is heterogeneous and contains some green monazite. It may have been cross-cut by a dark, uniform, 'sideritic' vein which pre-dates the monazite-strontianite vein. |
| 1993,P4(28) | K26 | Dark, medium grained (0.5 mm) carbonatite with some unknown white crystals but no observed monazite. |
| 1993,P4(29) | K27 | Dark, mineralised carbonatite with some unknown white crystals (apatite) but no observed monazite |
| 1993,P4(32) | K29 | Massive quartz cut by fenite veins |
| 1962,73(50) | G428 | Apatite-dolomite carbonatite (beforsite) which is finer grained than (58). Apatite are fractured but fresh. Magnetite is a major component, and there are occasional 450 µm crystals of perovskite. Blue Nb-rutile is also present, as are 100 um baddeleyite crystals. A vein of calcite cuts the rock and contains small, unidentified, acicular crystals. |
| 1962,73(58) | G1491F | Apatite-dolomite carbonatite (beforsite). Baddeleyite has an outer mantle of kimzeyite. There is relic olivine (now serpentine) |
| 1962,73(59) | G1326 | Apatite-dolomite carbonatite (beforsite) which has been phlogopitised. Can see alteration of apatite under CL and measure increase in REE in altered areas by EPMA. Contains corroded apatite in vein of alteration which has been altered to bastnäsite, strontianite, baryte fluorite and monazite. Major fresh apatite, magnetite and dolomite. Accessory zoned pyrochlore with higher Ce in the core and Sr at the edge. |
| 1962,73(77) | G464 | Siderititc dolomite/ankerite carbonatite patch in large northern intrusion. Plus a sharp contact with monazite-rich carbonatite. Good aggregates of green/colourless zoned euhedral monazite with strontianite and baryte with a little dolomite and calcite. A vein of 'sideritic' carbonatite penetrates the monazite-bearing carbonatite. Sphalerite grains are surrounded by smithsonite. |
| 1962,73(87) | G1160 | Dolomite carbonatite in which dolomite (60%) crystals are 400 µm to 105 µm diameter, monazite (20%) occurs as aggregates of euhedral and subhedral crystals and also single crystals. Largest monazites are 500 um. The monazite is zoned green and colourless in thin section. Altered areas have aggregates of 100 µm goyazite crystals (10%) around the monazite. Baryte (10%) 4 mm patches enclose monazite, and rare bastnäsite occurs. Many exsolved Fe oxide grains are seen in carbonate in thin section, but not observed on EPMA. |
| 1962,73(96) | G1327 | Manganiferous carbonatite with radiate apatite. Hexagonal pseudomorphs of carbonate and opaques, 200 um in diameter. Also contains quartz, baryte. Apatite tends to have more turbid cores and clearer rims, but no clear overgrowths (ie different to 131). There is some carbonate amongst the apatite. Quartz appears late. Baryte is not directly associated with the apatite. |
| 1962,73(100) | G1148 | Leucocratic carbonatite with patches of sideritic carbonatite. Anhedral green monazite (10%), strontianite. Intergrown apatite and goyazite (5%). Part of large carbonatite dyke |
| 1962,73(114) | G1492 | Monazite-quartz rock. Subhedral, 0.4–3.5 crystals of quartz (60%) enclose monazite crystals and have inclusions parallel to their C-axis. They contain fluid inclusions but they are probably secondary. Monazite (10%) euhedral crystals occur in baryte aas weel as quartz and are slightly pleiochroic green-pink. Goyazite (10%) occurs in turbid, 2mm, aggregates of euhedral 20 µm crystals. The crystals have oscillatory zoning of Sr vs La and Ce and 10–100 um inclusions of synchysite and bastnäsite. There is also anhedral goyazite in the aggregates as well as the euhedral crystals. Baryte crystals are 0.5-3 mm diameter and have fluid inclusions. |
| 1962,73(117) | G165 | Monazite quartz rock. Quartz (50%) crystals are 0.1-2 mm diameter and sub-anhedral. Monazite (10%) crystals are euhedral and 50-200 um in diameter, enclosed in baryte, quartz or goyazite. They are very pale green and often occurs as aggregates. Goyazite (15%) occurs as aggregates (up to 1cm) of 10-100 um rhombs which are turbid pink-brown and have inclusions of bastnäsite laths (1x20 to 50 um). The goyazite is zoned in La and Ce vs Sr. Baryte (15%) crystals are 400 um diameter and subhedral. Some have embayed margins. There are associated with monazite and goyazite. Bastnäsite occurs only as inclusions in goyazite. Monazite and florencite neighbour each other. |
| 1962,76(124) | G451 | Monazite-quartz-Fe oxide rock from outside the main complex |
| 1962,73(126) |  | Quartz-baryte-monazite-Fe oxide rock. |
| 1962,73(128) | G480 | Quartz druse rock with florencite from dyke 1 mile west of Kangankunde Hill. Quartz (60%) up to 1.5 mm, anhedral and encloses goyazites and opaques. Contains lots of minute inclusions often defining ghost crystals. Fe oxides and Ba, Mn oxides (30%) often 150 µm diameter but up to 450 µm. some are euhedral, occasionally with rhobohedral outlines and a 'textured' surface. Composition is variable. Described by Garson as a ‘rhombohedral carbonate’. Goyazite and intergrown apatite (10%) 50–200 um in rhombs. Two types of goyazite, one is more REE-rich. Textures are complex with either goyazite or apatite forming the majority of the grain. In PPL many grains have dark brown cores. Monazite is accessory phase as 30 µm grains with 2 wt.% ThO_2_, 1 wt.% UO_2_. Other accessory minerals are baryte and an Sn-Ni-sulphide grain |
| 1962,73(131) | G1175 | Quartz druse rock, rich in apatite. Subhedral (40%) quartz crystals, 50-450 µm diameter forming 1x4 mm patches. Apatite (40%) occurs in 1x4 mm aggregates of 200 um crystals which have turbid cores and clear euhedral rims. 20% of the rock comprises aggregates of opaque rhombs of Fe plus Mn and Si. Accessory minerals include Nb-rutile, xenotime and boulangerite. |
| 1962,73(133) | G1172 | Quartz-fluorite rock consisting of quartz (50%) and fluorite (50%). No REE, Sr or Ba were detected in the fluorite. Contains accessory strontianite and baryte. |
| 1968,P37(307) | | Quartz fenite containing a fenite vein. |
| SoS-168 | | Beforsite, consisting of dolomite, apatite, phlogopite and minor dolomite ocelli. |
| SoS-169 | | Beforsite, consisting of dolomite, apatite, phlogopite and minor dolomite ocelli. |
| SoS-170 | | Beforsite, consisting of dolomite, apatite, phlogopite and minor dolomite ocelli. |

*Field numbers for BM 1962, 73 (XXX) correspond to sample numbers in Garson and Campbell Smith (1965).

**Supplementary Table 2**: Whole-rock analyses of Kangankunde carbonatites

|  | **Apatite-dolomite carbonatites** | | | **REE-rich carbonatites** | | | | | | | | | | | | **REE-poor carbonatites** | | |
| --- | --- | --- | --- | --- | --- | --- | --- | --- | --- | --- | --- | --- | --- | --- | --- | --- | --- | --- |
| **BM 1993/1962** | **73 (50)** | **73 (58)** | **73 (59)** | **P4 (1)** | **P4 (7)** | **P4 (9)** | **P4 (14)** | **P4 (15)** | **P4 (19) vein** | **P4 (19) host** | **73 (77) vein** | **73 (77) host** | **73 (87)** | **73 (100)** | **GRC-6*** | **P4 (5)** | **P4 (28)** | **73 (96)** |
| SiO_2_ | 9.3 | 11.2 | 16.3 | 2.39 | 0.21 | 0.73 | 9.74 |  |  | 0.47 | 0.34 | 0.32 | 3.12 | 0.4 | 0.71 | 0.01 | 0.8 | 9.62 |
| TiO_2_ | 1.311 | 1.98 | 2.219 |  | 0.001 |  | 0.365 |  | 0.002 | 0.019 | 0.002 |  | 0.016 | 0.006 |  |  | 0.008 | 0.432 |
| Al_2_O_3_ | 1.87 | 2.26 | 3.74 | 0.05 | 0.05 | 0.04 | 1.94 | 0.08 | 0.09 | 0.43 | 0.08 | 0.03 | 0.72 | 0.51 | 0.04 | 0.04 | 0.27 | 0.6 |
| FeO_t_ | 10.8 | 14.1 | 10.9 | 4.09 | 5.29 | 3.7 | 12.1 | 5.65 | 4.02 | 7.58 | 1.61 | 8.3 | 4.74 | 2.77 | 2.42 | 7.94 | 13.3 | 4.32 |
| MnO | 0.55 | 0.63 | 1.24 | 1.39 | 2.1 | 1.46 | 2.4 | 2.16 | 1.58 | 1.61 | 0.72 | 0.31 | 1.71 | 1.06 | 0.93 | 3.45 | 5.86 | 0.61 |
| MgO | 9.92 | 10.9 | 12.8 | 9.45 | 12.6 | 8.88 | 9.42 | 1.18 | 14.3 | 10.2 | 5.34 | 14.1 | 12.2 | 6.72 | 7.07 | 14.8 | 9.74 | 1.77 |
| CaO | 36.6 | 35.2 | 15.7 | 16 | 18.9 | 21.5 | 22.7 | 30.9 | 25.4 | 30.9 | 9.27 | 26.6 | 20.8 | 15.2 | 12.70 | 24.1 | 26.3 | 38.3 |
| Na_2_O | 0.81 | 1.04 | 0.18 | 0.03 | 0.06 | 0.02 | 0.06 | 0.01 | 0.21 | 0.05 | 0.17 | 0.02 | 0.05 | 0.04 | 0.08 | 0.01 | 0.04 | 0.1 |
| K_2_O | 0.03 | 0.04 | 1.9 | 0.03 | 0.02 | 0.01 | 0.22 | 0.01 | 0.15 | 0.01 | 0.01 | 0.01 | 0.02 | 0.02 |  | 0.02 | 0.12 | 0.15 |
| BaO | 0.03 | 0.05 | 0.26 | 2.31 | 2.38 | 0.84 | 1.37 | 1.57 | 0.88 | 1.36 | 4.36 | 0.19 | 3.87 | 3.47 | 2.72 | 0.45 | 0.65 | 3.08 |
| SrO | 0.54 | 0.38 | 2.37 | 18.8 | 12.8 | 15.4 | 0.75 | 13.3 | 1.09 | 2.01 | 31.8 | 2.35 | 1.19 | 25.5 | 18.86 | 3.43 | 2.3 | 3 |
| H_2_O | 2.56 | 2.08 | 0.57 | 0.42 | 0.9 | 0.44 | 2.9 | 1.55 | 0.3 | 1.5 | 0.34 | 1.32 | 0.75 | 0.33 | na | 0.14 | 2.17 | 0.8 |
| P_2_O_5_ | 8.41 | 8.44 | 5.94 | 4.59 | 3.35 | 4.82 | 2.17 | 5.12 | 4.94 | 14.7 | 6.93 | 0.93 | 5.44 | 7.17 | 7.49 | 0.91 | 5.22 | 25.6 |
| CO_2_ | 14.5 | 10.2 | 22.7 | 25.3 | 29.5 | 27.4 | 31.5 | 26.1 | 39 | 27.2 | 14.9 | 38.6 | 32.3 | 20.2 | (27.2)* | 41.9 | 29.6 | 5 |
| F | 0.6 | 0.49 | 1.36 | 0.01 | 0.02 | 0.1 | 0.09 | 0.1 | 0.01 | 1.02 | 0.04 | 0.08 | 0.07 | 0.28 | 0.02 | 0.01 | 0.42 | 2.36 |
| Cl | 0.03 | 0.03 | 0.01 |  |  | 0.01 | 0.02 |  |  | 0.01 |  |  | 0.01 |  | na | 0.01 | 0.01 |  |
| SO_3_ | 0.01 | 0.03 | 0.02 | 1.12 | 1.15 | 0.35 | 0.45 | 0.55 | 0.4 | 0.3 | 1.3 | 0.1 | 0.3 | 1.81 | na | 0.23 | 0.08 | 1.47 |
| S | 0.02 | 0.01 |  |  |  |  |  |  |  |  |  | 0.11 |  | 0.11 | na | 0.13 |  |  |
| REE_2_O_3_ | 0.29 | 0.31 | 1.11 | 9.8 | 8.11 | 8.66 | 2.94 | 8.24 | 8.68 | 3.19 | 15.03 | 0.43 | 12.14 | 10.7 | 16.28 | 1.94 | 1.35 | 0.94 |
| Others | 0.25 | 0.28 | 0.19 | 0.09 | 0.24 | 0.18 | 0.37 | 0.11 | 0.23 | 0.39 | 0.15 | 2.35 | 0.2 | 0.28 | 0.14 | 0.39 | 0.39 | 0.21 |
| O=F,Cl,S | 0.27 | 0.22 | 0.57 | 0.01 | 0.01 | 0.04 | 0.04 | 0.04 | 0.03 | 0.43 | 0.02 | 0.09 | 0.03 | 0.17 | 0.01 | 0.07 | 0.18 | 0.99 |
| Total | 98.2 | 99.04 | 98.88 | 95.9 | 97.6 | 94.48 | 101.43 | 96.59 | 101.37 | 102.43 | 92.36 | 98.84 | 99.53 | 96.45 | 96.66 | 99.83 | 98.48 | 97.39 |
|  |  |  |  |  |  |  |  |  |  |  |  |  |  |  |  |  |  |  |
| Sc | 7.3 | 7.3 | 8.6 | 5.4 | 5.5 | 7.1 | 6.4 | 4.9 | 5.1 | 3.4 | 2.3 | 3.7 | 13.8 | 2.6 | 9 | 4.7 | 13.2 | 2.8 |
| Y | 138 | 133 | 49 | 46 | 83 | 65 | 43 | 33 | 33 | 55 | 58 | 18 | 101 | 104 | 79 | 16 | 31 | 103 |
| La | 489 | 509 | 2580 | 30300 | 21200 | 26000 | 6650 | 22000 | 24000 | 8500 | 38300 | 886 | 31000 | 27200 | 46300 | 4300 | 2540 | 1910 |
| Ce | 1020 | 1110 | 5050 | 41500 | 35600 | 36700 | 12400 | 34900 | 36900 | 13600 | 62800 | 1960 | 51600 | 45600 | 70400 | 8550 | 5680 | 3910 |
| Pr | 143 | 150 | 450 | 3050 | 2910 | 2830 | 1530 | 3010 | 3140 | 1150 | 6150 | 170 | 4640 | 4100 | 6250 | 826 | 649 | 422 |
| Nd | 523 | 585 | 1150 | 8360 | 8850 | 7810 | 3960 | 9420 | 9220 | 3600 | 19700 | 527 | 14800 | 13000 | 18000 | 2640 | 2310 | 1470 |
| Sm | 77 | 83 | 82 | 336 | 439 | 365 | 283 | 599 | 513 | 229 | 1040 | 37 | 1020 | 869 | 1190 | 138 | 178 | 121 |
| Eu | 25 | 27 | 20 | 62 | 83 | 67 | 55 | 112 | 95 | 45 | 190 | 8 | 212 | 147 | 192 | 25 | 32 | 28 |
| Gd | 79 | 83 | 50 | 98 | 142 | 113 | 107 | 192 | 161 | 102 | 272 | 27 | 381 | 315 | 283 | 51 | 69 | 74 |
| Tb | 47 | 55 | 44 | 2.1 | 5.2 | 0.7 | 43 | 9.3 | 1.4 | 26 |  | 39 | 1.5 |  | 25 | 25 | 47 | 17 |
| Dy | 44 | 46 | 21 | 31 | 45 | 37 | 30 | 37 | 33 | 31 | 58 | 14 | 82 | 79 | 49 | 15 | 24 | 32 |
| Ho | 6.9 | 7.5 | 4.2 | 6.4 | 7.9 | 7.5 | 4.8 | 6.8 | 6.7 | 5.5 | 7.8 | 3.3 | 11 | 11 | 5 | 3.9 | 4.6 | 6.3 |
| Er | 12.2 | 14 | 5.9 | 3.5 | 5.5 | 5.9 | 5.6 | 4.6 | 3.6 | 5.5 |  |  | 12 | 9 | 6.5 | 2.4 | 4.9 | 9.5 |
| Tm | na | na | na | na | na | na | na | na | na | na | na | na | na | na | 0.4 | na | na | na |
| Yb | 7.4 | 7.8 | 2.2 | 1 | 2.6 | 2.5 | 1.3 |  |  | 1.4 |  |  | 1.4 | 1.4 | 2 |  | 1.6 | 7.2 |
| Zn | 282 | 264 | 309 | 388 | 1360 | 1130 | 2570 | 554 | 1520 | 2640 | 829 | 18600 | 1130 | 1650 | 1030 | 2940 | 2680 | 1020 |
| Pb | 39 |  |  | 134 | 108 | 111 | 40 | 110 | 72 | 49 | 192 | 35 | 121 | 196 | 73 | 38 | 143 | 33 |
| Co | 16 | 24 | 22 | 4.6 | 4.1 | 2.7 | 11 |  | 7.2 | 4.6 | 3 | 12 |  | 2.9 | 1.5 | 7.1 | 5.4 | 9.6 |
| Cd |  |  |  | 8.5 | 1.6 | 15 | 2.2 | 1.5 | 11 | 15 | 2 | 45 | 3.5 | 14 | 29.2 | 1.9 |  |  |
| Ni |  |  |  |  | 200 |  |  |  |  |  |  | 49 |  |  |  |  |  |  |
| Cr | 5.2 | 7.9 | 6.6 |  |  | 11 | 8.6 |  | 6.8 | 11 |  |  |  |  |  |  |  | 12 |
| V | 65 | 67 | 34 | 19 | 19 | 31 | 53 | 35 | 38 | 43 | 11 | 27 | 67 | 17 | 30 | 37 | 59 | 46 |
| Be | 1.3 | 1.4 | 4.4 | 7.1 | 5.1 | 5.6 | 2.9 | 5.3 | 6.1 | 4.3 | 24.1 | 0.4 | 10.3 | 21.1 | na | 1.3 | 2 | 2.1 |
| Nb |  |  |  | 41 | 31 | 35 | 86 | 31 | 33 | 16 | 44 | 44 | 45 | 40 | 202 | 8 | 9 |  |
| Cu | 76 | 7.6 | 9.1 |  | 2.1 |  | 14 |  |  | 3.7 |  | 7.3 |  | 15 | 23 | 7.6 | 2.3 | 3.2 |
| Li | 27 | 43 | 65 | 13 | 10 | 13 | 12 | 14 | 11 | 8.3 | 10 | 2 | 18 | 13 | na | 16 | 14 | 6.6 |
| Zr | 1190 | 1350 | 800 | 41 | 43 | 18 | 61 | 61 | 78 | 277 | 18 | 23 | 68 | 123 | 47.3 |  | 164 | 367 |

Notes: Samples 1–32 prefixed with BM1993 (P4); 33–133 prefixed with BM1962 (73); 134–306 with SoS-; and 307 with BM1968 (P37). GRC-6* compositions from Verplanck et al. (2016), LOI data replace CO_2_. All other data analysed by V.K. Din and G.C. Jones; oxides in wt.%, trace elements in µg/g; FeO_t_ is total Fe as FeO; REE_2_O_3_ is the sum of the REE recalculated as oxides; ‘others’ denotes trace elements from the second part of the table; S quoted as SO_3_, except where S are concentrations are in excess of this value; blank cells are below detection, na = not analysed.

**Supplementary notes on fluid inclusion microthermometry:**

There are fluid inclusions in some carbonate crystals (e.g. BM 1962, 73, (77)). They are up to 40 µm in diameter and have vapour bubbles occupying about 30% of the inclusion. Similarly, baryte contains fluid inclusions of 10–30 µm in diameter (e.g. BM 1962, 73 (103) and (122)). There are some daughter minerals present and the inclusions have low/no CO_2_ contents. The vapour bubble makes up approximately 30% of the inclusion.

In both cases, the fluid inclusions are likely to be secondary in nature. Inclusions follow trails, cross-cutting minerals, and are simple, low-salinity, liquid-vapour type inclusions (Supplementary Figure 1). They are, therefore, not informative with regard to the formation temperature of the REE mineralisation at Kangankunde.


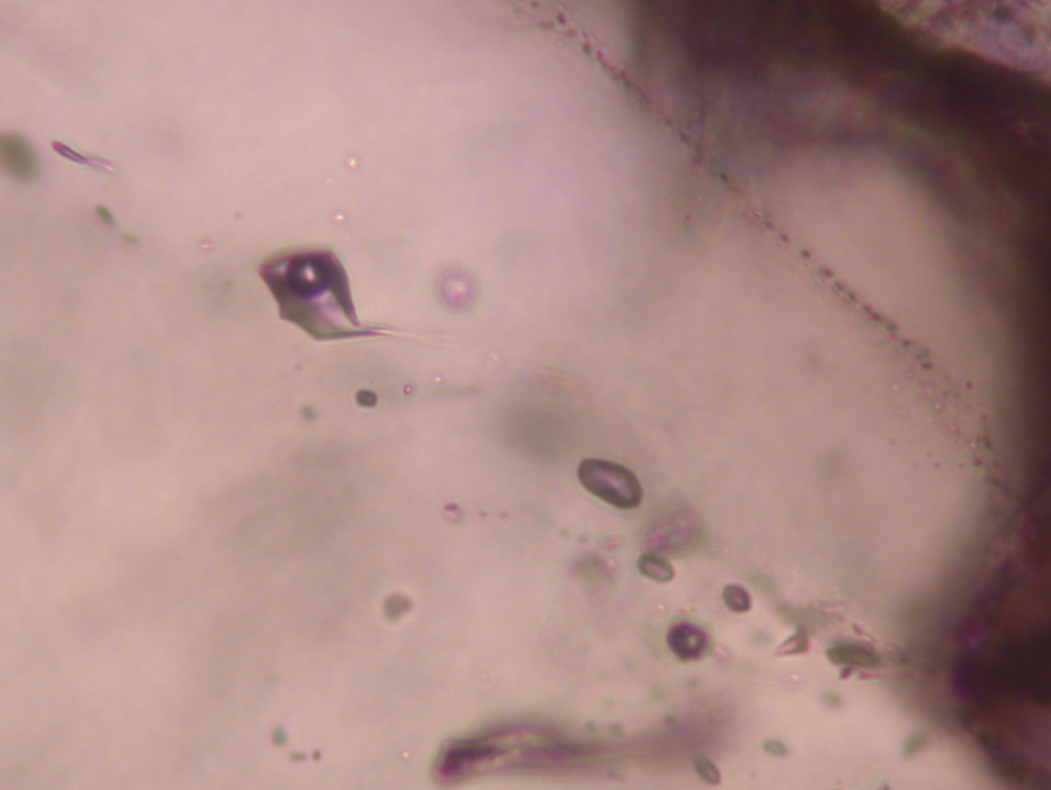


Supplementary Figure 1: secondary fluid inclusion trail in carbonate. Sample BM1962 73 (103). Image courtesy of Martin Smith.
